# Which Interventions Offer Best Value for Money in Primary Prevention of Cardiovascular Disease?

**DOI:** 10.1371/journal.pone.0041842

**Published:** 2012-07-23

**Authors:** Linda J. Cobiac, Anne Magnus, Stephen Lim, Jan J. Barendregt, Rob Carter, Theo Vos

**Affiliations:** 1 School of Population Health, The University of Queensland, Herston, Queensland, Australia; 2 Deakin Health Economics, Strategic Research Centre – Population Health, Deakin University, Burwood, Victoria, Australia; 3 Institute for Health Metrics and Evaluation, Seattle, Washington, United States of America; Erasmus University Rotterdam, Netherlands

## Abstract

**Background:**

Despite many decades of declining mortality rates in the Western world, cardiovascular disease remains the leading cause of death worldwide. In this research we evaluate the optimal mix of lifestyle, pharmaceutical and population-wide interventions for primary prevention of cardiovascular disease.

**Methods and Findings:**

In a discrete time Markov model we simulate the ischaemic heart disease and stroke outcomes and cost impacts of intervention over the lifetime of all Australian men and women, aged 35 to 84 years, who have never experienced a heart disease or stroke event. Best value for money is achieved by mandating moderate limits on salt in the manufacture of bread, margarine and cereal. A combination of diuretic, calcium channel blocker, ACE inhibitor and low-cost statin, for everyone with at least 5% five-year risk of cardiovascular disease, is also cost-effective, but lifestyle interventions aiming to change risky dietary and exercise behaviours are extremely poor value for money and have little population health benefit.

**Conclusions:**

There is huge potential for improving efficiency in cardiovascular disease prevention in Australia. A tougher approach from Government to mandating limits on salt in processed foods and reducing excessive statin prices, and a shift away from lifestyle counselling to more efficient absolute risk-based prescription of preventive drugs, could cut health care costs while improving population health.

## Introduction

Despite many decades of declining mortality rates in the Western world [1], [2], cardiovascular disease (CVD) remains the leading cause of death worldwide [3]. In countries such as the United States (US), United Kingdom (UK) and Australia, the majority of cardiovascular burden could be prevented by better addressing key risks, such as blood pressure and cholesterol levels [4]–[6].

Many countries have already developed guidelines and implemented interventions for primary prevention of CVD. Most guidelines recommend lifestyle behaviour-change approaches as a first line strategy, with blood pressure-lowering and/or cholesterol drugs for those at highest risk [7]. In England, for example, the new Vascular Check program aims to screen all adults aged between 40 and 74 years, targeting those at risk with a combination of lifestyle interventions, blood pressure-lowering drugs and statin drugs, where indicated [8]. Some countries have also implemented community- or population-wide interventions; many community-level heart health programs were run between the 1970s and 1990s [9], and countries, such as Finland and the UK, have also established long-term programs to reduce population dietary salt levels [10].

With rising health care costs, it is vital that countries combine intervention strategies that will achieve maximum improvement in cardiovascular health at lowest cost to the health sector. Cost-effectiveness analyses for the WHO-CHOICE program in 2000, showed that a combination of beta-blocker, diuretic, statin and aspirin could be cost-effective, if provided to everyone with at least a 5% probability of a cardiovascular event in the next ten years, in regions with low child and adult mortality (e.g. UK, US and Australia) [11]. Newer evidence on drug efficacy [12], [13], including alternative blood pressure-lowering drugs such as calcium channel blockers and ACE inhibitors, the changing price of statins [14]–[16], and increasing doubts about the use of aspirin in primary prevention [17], [18], however, may mean that this is no longer the optimal strategy for intervention. More recently, England’s Department of Health has estimated that the new Vascular Check program combining lifestyle intervention with drugs (statins, ACE-inhibitors and calcium channel blockers) for those at highest risk will be highly cost-effective (£3000/QALY ≡ A$6,700/QALY [19]) [8], [20]. Although this has not been compared with the cost-effectiveness of population-wide strategies, analyses from WHO-CHOICE, Argentina, Vietnam, the UK, US and Australia, have found that population-wide strategies, particularly salt reduction programs are likely to be even more cost-effective [11], [21], [22] and potentially even cost-saving [23]–[25].

Australia is currently re-visiting its guidelines for primary prevention of cardiovascular disease. In this research we evaluate the optimal mix of lifestyle, pharmaceutical and population-wide interventions from an Australian health sector perspective. We also evaluate the current practice of blood pressure and cholesterol intervention in Australia, and examine this against the optimal mix, to quantify the potential for improving population health and reducing health sector expenditure.

## Methods

### Ethics Statement

The study was approved by the Behavioural & Social Sciences Ethical Review Committee of the University of Queensland in accordance with the National Health and Medical Research Council guidelines (Clearance no. 2004000796). The study was based on analysis of publically available data. It did not involve human participants or require informed consent.

**Table 1:** Intervention costs and effects.

Intervention	Annual cost per person*	Measure of effect	Effect size	Sources**
Thiazide diuretic	$71	RR IHD RR stroke	0.86 (0.06) 0.62 (0.05)	[13], [37], [38]
Beta-blocker	$106	RR IHD RR stroke	0.89 (0.06) 0.83 (0.07)	[13], [37], [38]
Calcium channel blocker	$218	RR IHD RR stroke	0.85 (0.04) 0.66 (0.04)	[13], [37], [38]
ACE inhibitor	$212	RR IHD RR stroke	0.83 (0.03) 0.78 (0.07)	[13], [37], [38]
Aspirin	$40	RR IHD RR stroke (isch.) RR stroke (haem.) RR GI bleed	0.82 (0.04) 0.86 (0.07)1.32 (0.19) 1.54 (0.13)	[37], [71]–[73]
Statin	Aust.: $687 NZ: $19	RR IHD RR stroke	0.70 (0.05) 0.81 (0.06)	[12], [37], [39], [64], [66]
Phytosterol margarine	$258 ($38)	Total cholesterol	7.5% (1.9%)	[42], [43], [74], [75]
Dietary advice	Yr 1: $132 ($213) Yr 2+: $86 ($39)	Systolic BP Total cholesterol	1.6% (0.4%) 3.1%(1.2%)	[47], [76]
Lifestyle program	Yr 1: $257 ($152) Yr 2+: $172 ($58)	Systolic BP Total cholesterol	2.6% (0.5%) 3.3%(0.6%)	[47], [48], [77]
Community heart health program	Yr 1: $2.37 ($0.47) Yr 2+:$1.60 ($0.32)	Systolic BP Total cholesterol	2.5% (0.7%)−0.51% (0.6%)	[9], [44]
Mandatory salt reduction	$0.81 ($0.08)	mgNa/day men mgNa/day women	10.6 (0.74) 7.3 (0.53)	[45], [46], [49], [78]
Voluntary salt reduction (current practice)	$0.49 ($0.05)	mgNa/day men mgNa/day women	0.50 (0.03) 0.34 (0.02)	[45], [49]
Lipid-lowering (current practice)	$683	RR IHD RR stroke	0.70 (0.05) 0.81 (0.06)	[12], [37]
BP-lowering (current practice)	$170	RR IHD RR stroke	0.85 (0.04) 0.70 (0.05)	[13], [37], [40]

NB. Values are mean and standard error, unless otherwise stated. BP – blood pressure; GP – general practitioner; NZ – New Zealand; RR – relative risk; IHD – ischaemic heart disease.

* All costs are adjusted to 2008 Australian dollars using consumer price indices [79], health sector inflators [64] and purchasing power parities [19] where relevant.

** Table A2 provides further detail of sources and assumptions underlying the measurement of intervention costs and effects.

We evaluate cost-effectiveness of interventions to prevent CVD in the 2008 Australian population of 35 to 84 year olds who have not previously experienced a CVD event (defined as angina, myocardial infarction or stroke). We include two interventions targeting the whole population, a community heart health program and mandatory reduction of salt in the manufacture of breads, margarines and cereals; six interventions targeting those at increased risk of disease with pharmacological agents, diuretics, ACE inhibitors, calcium channel blockers, beta-blockers, statins and aspirin; and three interventions targeting those at increased risk of disease with interventions to change behaviour, dietary advice from a doctor or dietitian, referral to a more intensive lifestyle program with specialised counselling, and advice from a doctor to switch to phytosterol-enriched margarine. We also model the current practice coverage of these interventions for primary prevention of CVD in Australia. Key components of current practice include voluntary (rather than mandatory) reduction of salt in breads, margarines and cereals; dietary advice from a general practitioner (GP); and blood pressure- and cholesterol-lowering drug therapies.

### Intervention Uptake and Adherence

Two of the interventions, the community heart health program and reduction of salt in processed foods, are delivered to the whole population. We assume the average population effect of these interventions is sustained with ongoing delivery of the interventions.

All other interventions are delivered in primary care. We determine the annual number of 35 to 84 year olds visiting a GP from an Australian GP sample registration system [26] and determine GP participation in CVD risk assessment from GP involvement in Australia’s Practice Incentives Program [27]. Eligibility for preventive therapy is based on an individual’s risk of a CVD event over the next five years [28], divided into three levels of risk: ≥15%, 10–14% and 5–9%.

We determine the probability of an event using the Framingham risk prediction equation [29]. The equation is calibrated for the Australian population using probabilities of a first-ever ischaemic heart disease or stroke event (derived from Australian hospital and mortality databases [30], [31], the Perth MONICA study [32] and the NEMESIS [33] study) and individual-level data from Australia’s AusDiab 1999–2000 data set [34]. The AusDiab data used in the Framingham equation include age, sex, smoking status, total cholesterol level, high density lipoprotein cholesterol level and diabetes status, for everyone who has not had an ischaemic heart disease or stroke event. Rather than altering the parameters of the Framingham risk prediction equation, we scale the predicted risk to fit the actual observed risk in the Australian population, by age and sex. The predicted risk is then used to determine: (a) the numbers of Australians who are eligible for each intervention (or intervention combination); and (b) their initial cardiovascular disease risk, relative to the mean risk in the population, by age and sex.

Of those patients eligible to receive intervention, we assume that 40% will no longer be adherent after 12 months, based on rates of discontinuation with blood pressure-lowering and cholesterol-lowering therapies in Australia [35], [36].

### Intervention Costs and Effects

The modelled measures of intervention costs and effects are summarised in Table 1 with further detail available in Table A2 of the supplementary text.

Drug costs are based on prices in Australia’s Pharmaceutical Benefits Scheme (PBS) list of tax-subsidised drugs [37]. Each cost is estimated as an average across the class, weighted by the mix of scripts provided in 2008 for an equivalent standard dose [38], [39] (e.g. 40 mg/day simvastatin, 20 mg/day atorvastatin, 10 mg/day rosuvastatin and 80 mg/day pravastatin) [18], [38]. The costs of lipid-lowering and blood pressure-lowering drugs used in current practice are derived from recorded PBS cost data from the baseline year of 2008, using general practice data [40] to estimate the mix of blood pressure-lowering drugs in preventive practice. Each intervention also includes the costs of initial and follow-up GP visits and blood tests for measurement of lipid levels and on-going monitoring (e.g. monitoring of renal function for patients on ACE inhibitors), based on the listed prices in Australia’s Medicare Benefits Schedule (MBS) [41].

The cost of phytosterol-enriched margarine is derived from a price survey of all available products available at the two major Australian supermarket chains, assuming that each product contains the Australian standard concentration of plant sterols (82 g per kg of margarine [42]) and that 3.4 g of plant sterols are required each day to achieve a beneficial effect [43].

The cost of the community heart health program is based on the bottom-up costing of the Hartslag Limburg cardiovascular prevention project [44].

The cost of the current voluntary program for salt reduction in breads, margarines and cereals is derived from the proportion of products participating in the current Heart Foundation program [45] and the annual fee per product (C. Colyer, Heart Foundation; personal communication, 18 June 2009). Costs of legislative changes and enforcement for the mandatory program are derived from World Health Organisation unit costs (www.who.int/choice/costs/en/) in Australia and resource use [46].

**Table 2:** Effectiveness of the interventions for primary prevention of CVD, when evaluated individually against the partial null ‘do nothing’ strategy and when evaluated as an addition to the most cost-effective package.

Intervention and target group	Health gain of intervention when implemented individually (DALYs)*	Health gain of intervention when added to the package (DALYs)*
Mandatory salt limits (all risk levels)	80,000 (60,000 to 100,000)	80,000 (60,000 to 100,000)
Diuretic (≥15% risk)	39,000 (22,000 to 59,000)	38,000 (22,000 to 58,000)
Diuretic (10–14% risk)	40,000 (23,000 to 61,000)	39,000 (22,000 to 59,000)
Diuretic (5–9% risk)	77,000 (43,000 to 120,000)	75,000 (42,000 to 110,000)
Ca channel blocker (≥15% risk)	37,000 (24,000 to 54,000)	28,000 (18,000 to 41,000)
Ca channel blocker (10–14% risk)	39,000 (25,000 to 55,000)	29,000 (18,000 to 42,000)
ACE inhibitor (≥15% risk)	31,000 (18,000 to 47,000)	20,000 (12,000 to 30,000)
Ca channel blocker (5–9% risk)	74,000 (47,000 to 110,000)	56,000 (34,000 to 81,000)
ACE inhibitor (10–14% risk)	32,000 (19,000 to 49,000)	21,000 (13,000 to 31,000)
ACE inhibitor (5–9% risk)	62,000 (36,000 to 95,000)	40,000 (24,000 to 61,000)
Statin (≥15% risk)	41,000 (24,000 to 62,000)	25,000 (15,000 to 38,000)
Comm. heart program (all risk levels)	3,000 (1,500 to 4,700)	2,600 (1,300 to 4,000)
Statin (10–14% risk)	43,000 (25,000 to 65,000)	27,000 (16,000 to 40,000)
Statin (5–9% risk)	85,000 (50,000 to 130,000)	51,000 (30,000 to 77,000)
Dietary advice (≥15% risk)	180 (110 to 280)	82 (46 to 130)
Dietary advice (10–14% risk)	190 (110 to 290)	86 (48 to 140)
Dietary advice (5–9% risk)	370 (210 to 580)	160 (91 to 270)
Phytosterol (≥15% risk)	160 (82 to 260)	80 (38 to 130)
Phytosterol (10–14% risk)	170 (86 to 270)	84 (40 to 140)
Phytosterol (5–9% risk)	330 (170 to 540)	160 (77 to 270)
Aspirin (≥15% risk)	19,000 (7,200 to 33,000)	*Not included in optimal package* **
Aspirin (10–14% risk)	20,000 (7,700 to 35,000)	*Not included in optimal package* **
Aspirin (5–9% risk)	39,000 (16,000 to 68,000)	*Not included in optimal package* **
Beta-blocker (≥15% risk)	21,000 (5,200 to 39,000)	*Not included in optimal package* **
Beta-blocker (10–14% risk)	22,000 (5,400 to 40,000)	*Not included in optimal package* **
Beta-blocker (5–9% risk)	42,000 (10,000 to 79,000)	*Not included in optimal package* **
Lifestyle program (≥15% risk)	250 (160 to 360)	*Not included in optimal package* **
Lifestyle program (10–14% risk)	270 (170 to 380)	*Not included in optimal package* **
Lifestyle program (5–9% risk)	520 (330 to 740)	*Not included in optimal package* **

* Values are mean and 95% uncertainty interval, rounded to two significant figures. DALY – Disability-adjusted life year.

** Intervention not included in the optimal package because a more cost-effective alternative is available.

**Table 3:** Cost-effectiveness of the interventions for primary prevention of CVD, when evaluated individually against the partial null ‘do nothing’ strategy and when evaluated as an addition to the most cost-effective package.

Intervention and target group	Cost-effectiveness of intervention when implemented individually ($/DALY)*	Cost-effectiveness of intervention when added to the package ($/DALY)*
Mandatory salt limits (all risk levels)	Dominant (Dominant to Dominant)	Dominant (Dominant to Dominant)
Diuretic (≥15% risk)	Dominant (Dominant to $5,600)	Dominant (Dominant to $5,600)
Diuretic (10–14% risk)	$2,000 (Dominant to $10,000)	$2,000 (Dominant to $10,000)
Diuretic (5–9% risk)	$5,800 (Dominant to $16,000)	$5,800 (Dominant to $16,000)
Ca channel blocker (≥15% risk)	$7,900 ($3,300 to $14,000)	$7,900 ($3,300 to $14,000)
Ca channel blocker (10–14% risk)	$12,000 ($6,700 to $20,000)	$12,000 ($6,700 to $20,000)
ACE inhibitor (≥15% risk)	$10,000 ($4,800 to $21,000)	$10,000 ($4,800 to $21,000)
Ca channel blocker (5–9% risk)	$19,000 ($12,000 to $29,000)	$19,000 ($12,000 to $29,000)
ACE inhibitor (10–14% risk)	$15,000 ($8,400 to $28,000)	$15,000 ($8,400 to $28,000)
ACE inhibitor (5–9% risk)	$23,000 ($14,000 to $40,000)	$23,000 ($14,000 to $40,000)
Statin (≥15% risk)	$28,000 ($18,000 to $46,000)	$28,000 ($18,000 to $46,000)
Comm. heart program (all risk levels)	$44,000 ($19,000 to $100,000)	$44,000 ($19,000 to $100,000)
Statin (10–14% risk)	$36,000 ($25,000 to $59,000)	$36,000 ($25,000 to $59,000)
Statin (5–9% risk)	$51,000 ($37,000 to $81,000)	$51,000 ($37,000 to $81,000)
Dietary advice (≥15% risk)	$1,000,000 ($610,000 to $2,400,000)	$1,000,000 ($610,000 to $2,400,000)
Dietary advice (10–14% risk)	$1,100,000 ($730,000 to $3,000,000)	$1,100,000 ($730,000 to $3,000,000)
Dietary advice (5–9% risk)	$1,400,000 ($920,000 to $3,900,000)	$1,400,000 ($920,000 to $3,900,000)
Phytosterol (≥15% risk)	$3,200,000 ($1,900,000 to $5,900,000)	$3,200,000 ($1,900,000 to $5,900,000)
Phytosterol (10–14% risk)	$3,900,000 ($2,400,000 to $7,300,000)	$3,900,000 ($2,400,000 to $7,300,000)
Phytosterol (5–9% risk)	$4,900,000 ($3,000,000 to $9,300,000)	$4,900,000 ($3,000,000 to $9,300,000)
Aspirin (≥15% risk)	$1,800 (Dominant to $18,000)	*Not included in optimal package* **
Aspirin (10–14% risk)	$3,500 (Dominant to $24,000)	*Not included in optimal package* **
Aspirin (5–9% risk)	$8,300 (Dominant to $34,000)	*Not included in optimal package* **
Beta-blocker (≥15% risk)	$10,000 ($1,100 to $74,000)	*Not included in optimal package* **
Beta-blocker (10–14% risk)	$15,000 ($3,300 to $94,000)	*Not included in optimal package* **
Beta-blocker (5–9% risk)	$22,000 ($7,700 to $130,000)	*Not included in optimal package* **
Lifestyle program (≥15% risk)	$1,400,000 ($960,000 to $2,500,000)	*Not included in optimal package* **
Lifestyle program (10–14% risk)	$1,600,000 ($1,100,000 to $3,200,000)	*Not included in optimal package* **
Lifestyle program (5–9% risk)	$2,100,000 ($1,400,000 to $4,100,000)	*Not included in optimal package* **

* Cost-effectiveness ratios are median and 95% uncertainty interval, rounded to two significant figures. Where the ratio is *Dominant*, the intervention is cost-saving.

** Intervention not included in the optimal package because a more cost-effective alternative is available.

The costs of dietary advice from a GP or dietitian and costs of participation in a lifestyle program are based on Australian Government costs for GP [41], dietitian [47] and/or exercise physiologist [48] attendance and estimates of the number of initial and follow-up visits.

Measures of intervention efficacy are based on meta-analyses of relevant randomised controlled trials, with the exception of voluntary and mandatory salt reduction, where program effectiveness is determined from a New Zealand study of the sodium reduction program [45] and current Australian data on consumption of breads, margarines and cereals [49] (Table 1 A2). For interventions that measure outcomes as a change in blood pressure or cholesterol (e.g. dietary advice, phytosterol margarine), reductions in relative risks of ischaemic heart disease and stroke are derived from the proportional changes found in meta-analyses of blood pressure-lowering and statin drug trials. A 1% reduction in systolic blood pressure is associated with 3.4% reduction in relative risk of ischaemic heart disease and 6.3% reduction in relative risk of stroke; and a 1% reduction in total cholesterol is associated with 1.8% reduction in relative risk of ischaemic heart disease and 0.80% reduction in relative risk of stroke [13], [50], [51]. For the salt interventions, a change in blood pressure is first derived, by age and sex, using the relationships between sodium and systolic blood pressure derived by Law et al. [52]. The efficacy of interventions involving combinations of interventions (e.g. a statin and a diuretic) are determined multiplicatively [53] (e.g. using the intervention effect data in Table 1, the relative risk of ischaemic heart disease with a combination of statin and diuretic is 0.70×0.86 = 0.602). For the interventions that have an effect on salt, blood pressure or cholesterol, the reductions in relative risks of disease are first derived, before being combined with other interventions multiplicatively.

**Figure 1:**
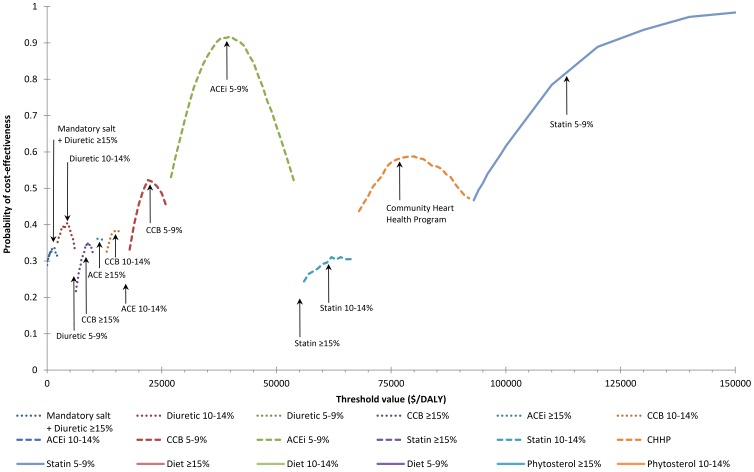
The cost-effectiveness acceptability frontier, shown for values of the cost-effectiveness threshold up to $150,000/DALY. Addition of the interventions that are not visible on the graph, is not optimal until much higher cost-effectiveness thresholds (dietary advice above $2.4 million/DALY and phytosterol margarine above $6.7 million/DALY).

**Figure 2:**
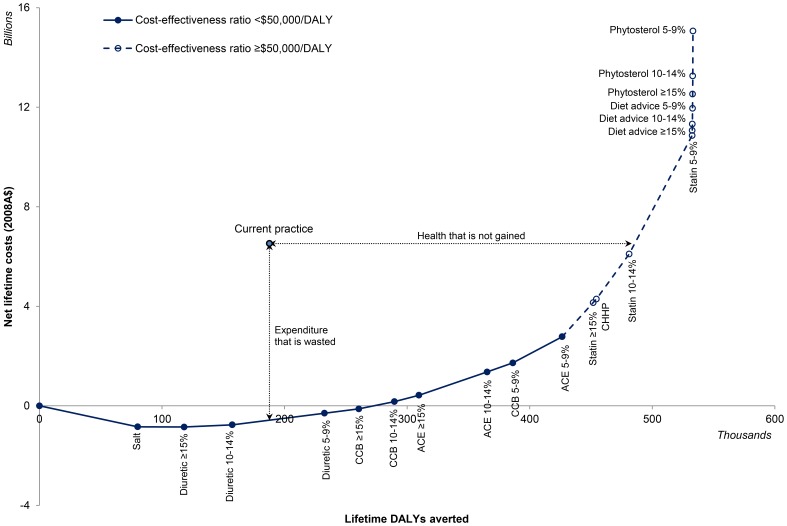
The cost-effectiveness of current practice and the optimal intervention pathway (NB. CCB – calcium channel blocker; ACEi – ACE inhibitor; CHHP – community heart health program). Interventions are added to the mix in order of cost-effectiveness, thus the pathway reflects the efficiency frontier. The pathway is shown as a solid line where the incremental cost-effectiveness of adding an intervention to the mix is under the cost-effectiveness threshold of $50,000/DALY, and shown as a dashed line where the addition of the next intervention is not cost-effectiveness (i.e. it exceeds the threshold of $50,000/DALY).

**Table 4:** Lifetime costs and health gain of the current practice for CVD prevention and of the most cost-effective package of interventions under different discounting and costing assumptions.

	Current practice	Cost-effective package*	Cost-effective package* + lower cost statins**
**Lifetime health gain (Thousands DALYs)**	190 (140 to 240)	430 (310 to 570)	530 (370 to 710)
**Intervention cost to government (2008A$billion)**	$7.1 ($5.7 to $8.5)	$5.5 ($3.9 to $7.3)	$6.3 ($4.5 to $8.4)
**Intervention cost to patients (2008A$billion)**	$1.6 ($1.3 to $1.9)	$2.0 ($1.4 to $2.7)	$2.1 ($1.5 to $2.9)
**Disease treatment costs averted (2008A$billion)**	−$2.2 (−$3.0 to −$1.5)	−$4.8 (−$6.9 to −$3.0)	−$6.1 (−$9.0 to −$3.8)
**Net lifetime cost (2008A$billion)**	$6.5 ($5.1 to $8.0)	$2.8 ($1.1 to $4.6)	$2.3 ($0.51 to $4.3)

* Cost-effective package includes population-wide mandatory limits on salt in breads, margarines and cereals, and a mix of diuretic, calcium channel blocker and ACE inhibitor drugs for everyone with at least 5% risk of a CVD event in the next five years.

** Statins provided for everyone with at least 5% risk of a CVD event in the next five years, at an annual cost of $18.25 (equivalent to the current price in New Zealand).

### Cost-effectiveness Modelling

Cost-effectiveness analysis is carried out using the ‘generalised cost-effectiveness analysis’ approach developed for the World Health Organisation [54], in which all interventions (including current practice) are evaluated against a theoretical ‘do nothing’ (i.e. do none of the interventions of interest in the analysis) comparator. This approach allows explicit estimation of the cost-effectiveness of current practice, it avoids artificially making an intervention look more favourable if compared against inefficient current practice, and it allows the optimal mix of interventions to be evaluated. We back-calculate disease rates under the ‘do nothing’ scenario using the same parameters of intervention effectiveness, adherence and costs that are used in the cost-effectiveness analyses. Hence, when current practice is modelled from the ‘do nothing’ scenario, the model reproduces the levels of disease currently observed. Current use of CVD preventive therapies is derived from AusDiab [34] and general practice data [40], [55].

**Figure 3:**
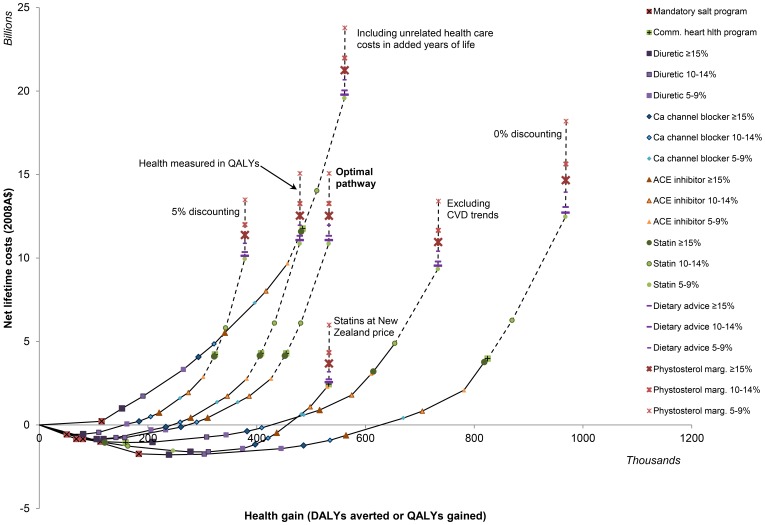
Sensitivity of the optimal pathway to increased and decreased discounting (5% and 0%), to the addition of other non-cardiovascular health care costs in added years of life, the measurement of health gain in QALYs rather than DALYs, and to a reduction in the cost of statin drugs to the much lower price in New Zealand (NB. the order of interventions is altered only by the reduction in statin price, with statins becoming a more cost-effective intervention option than the blood pressure-lowering drugs).

We use a discrete time Markov model to simulate costs and health outcomes for the population that is eligible for each intervention (or intervention combination), in five-year age and sex cohorts. The Markov model has four primary health states, with transition rates capturing probabilities of incidence and case fatality for fatal and non-fatal IHD and stroke events. Probabilities of gastrointestinal (GI) bleeds and hemorrhagic stroke are taken into account as side-effects of aspirin therapy. Rates are derived from Australian hospital and mortality databases [30], [31], the Perth MONICA study [32] and the NEMESIS [33] study. Trends are incorporated to capture underlying changes in IHD and stroke incidence and case fatality over time [56]. Further details of the modelling methods and inputs are provided in Text S1 and Text S2.

The total years of life lived by the population, both with and without intervention, are adjusted for time spent in ill health using utility or disability weights that capture the average quality of life or ‘disability’ experienced at each age and sex, with or without ischaemic heart disease, stroke or a GI bleed. This weighting process can be carried out using utility weights to derive quality-adjusted life years (QALYs), or using disability weights to derive the loss of health-related quality of life captured in disability-adjusted life years (DALYs). Although similar survey techniques are used to elicit health state preferences for both utility and disability weights, health state preferences for the QALY weights are typically elicited from surveys of patients or the general population while health state preferences for the DALY weights have been elicited from expert panels. A comparison of the utility and disability weights for ischaemic heart disease, stroke, GI bleeds and all other ‘background’ disability can be found in Text S1. In these analyses, we base our results on health gain measured in DALYs, but since there is debate about when QALYs or DALYs are the superior measure [57], we also evaluate cost-effectiveness in QALYs to determine the impact (if any) of the QALY or DALY choice on cost-effectiveness results.

Using Markov model predictions of the years of life lived and time spent in ill health, we simulate costs of treating IHD, stroke and GI bleed events. Annual costs in the initial year of illness and in subsequent years are determined from hospital in-patient costs [58], out-of-hospital expenses [59] and NEMESIS data for stroke [60]. All costs are adjusted to Australian dollars in the year 2008 using health system deflators [61].

We simulate costs and health outcomes over time until everyone in the population has died. All future costs and health outcomes are discounted at a rate of 3% [62]. Cost-effectiveness ratios are then evaluated in Australian dollars per DALY (or QALY) for the year 2008. In multivariate probabilistic uncertainty analysis, using Monte Carlo simulation, we derive 95% uncertainty intervals for all outcome measures and determine the probability of each intervention being cost-effectiveness against a cost-effectiveness threshold of $50,000/DALY [63].

## Results

Mandating more moderate use of salt in breads, margarines and cereals is easily the most effective (Table 2) and cost-effective (Table 3) strategy for primary prevention of CVD; it produces the biggest improvements in population health, and can save money for the health sector. The blood pressure-lowering drugs, including diuretics, calcium channel blockers and ACE inhibitors, are also cost-effective. If provided to people with at least 5% risk of a cardiovascular event in the next five years, these drugs can improve health for less than $50,000 per DALY.

Blood pressure-lowering with beta-blockers, although cost-effective, has a lower probability of improving population health than diuretic, calcium channel blocker and ACE inhibitor options, and would not be recommended in preference to these three readily available and cost-effective drugs. Aspirin, also cost-effective on average, has a much higher probability of causing harm than the other drugs evaluated; if other more cost-effective drugs are first provided, the potential health benefits of aspirin are reduced, and it is no longer a cost-effective strategy for primary prevention of CVD.

No other interventions represent good value for money (Figure 1). Statin drugs, including off-patent simvastatin, are currently very expensive in Australia and a community heart health program can achieve only small improvements in population health. These interventions, although cost-effective if implemented as isolated strategies, are not cost-effective if other more cost-effective strategies (mandatory salt reduction and blood pressure-lowering drugs) are first provided. The behaviour change interventions, including dietary advice, participation in a lifestyle program and switching to phytosterol-enriched margarine, can achieve only small improvements in population health and are least cost-effective of all the primary prevention strategies. Adding any of these interventions to the prevention package is very bad value for money at more than $1 million per DALY.

The current practice combination of blood pressure-lowering drugs, statin drugs, dietary advice and voluntary participation of food manufacturers in limiting salt use in processed foods, is inefficient compared to the optimal approach of mandating more moderate use of salt and providing diuretics, calcium channel blockers and ACE inhibitors for everyone with at least 5% cardiovascular risk (Figure 2). Providing the optimal package of interventions could reduce current health care expenditure of the Australian Government by $3.7 billion, while achieving more than double the improvements in population health, over the lifetime of the population (Table 4).

Reducing the costs of statin drugs would produce even greater benefits (Figure 3). With a reduction to the current price in New Zealand, statins would be a very cost-effective addition to the optimal package (more cost-effective than the blood pressure-lowering options). With the addition of cheaper statin drugs, the optimal intervention package could reduce current health care expenditure by $4.2 billion and achieve triple the population health that is achieved with current intervention choices (Table 4).

A number of factors influence the total costs and health gain of the optimal package of interventions, including discount rate, addition of other health care costs in added years of life, CVD trends and measurement of health in QALYs rather than DALYs, but these factors do not influence the order of interventions in the pathway (Figure 3 and Text S3). The optimal intervention package of mandatory limits on salt, diuretics, calcium channel blockers and ACE inhibitors, which is determined by reference to the $50,000/DALY threshold, is unchanged under all scenarios. However, two interventions, the community heart health program and the addition of statin drugs for everyone at 10 to 15% CVD risk, only just exceed the $50,000 per DALY threshold, and under assumptions that improve intervention cost-effectiveness, including lowering discount rates and ignoring current downward CVD trends, these two interventions would be included in the optimal intervention package The individually-targeted behaviour change interventions, though, are not cost-effectiveness under any scenario evaluated.

## Discussion

To achieve best value for money in the primary prevention of CVD, the Australian government must take a tougher approach in mandating limits on salt in processed foods (bread, margarine and cereal), and fund a combination of diuretic, calcium channel blocker, ACE inhibitor and (low cost) statin drugs for everyone found to have at least a 5% five-year risk of CVD when visiting their local GP. If implemented in Australia, this package of interventions could achieve a three-fold improvement in current population health and reduce current lifetime health care expenditure by $4.2 billion (Australia’s total health care expenditure is around $100 billion annually [64]). Current recommendations for lifestyle behaviour-change interventions as a first-line strategy for CVD prevention should be reconsidered; these interventions are poor value for money, achieving only trivial gains in population health at a very high cost.

Our findings are robust to modelling assumptions around discount rate, inclusion of other non-CVD health care costs in added years of life, and choice of health metric (DALY versus QALY), but are sensitive to drug price. It is likely, therefore, that the Australian cost-effectiveness results will broadly reflect cost-effectiveness of primary prevention strategies in other countries with similar epidemiological and health system characteristics (e.g. United Kingdom and New Zealand), with the exception of the results on statin drugs. Australia currently pays around five times the average price paid for statin drugs in other OECD countries [14], and at this price they are not a cost-effective addition to the intervention package. Australian legislative changes in November 2010 [65] will ensure a 16% cut in the price of the two most expensive statins (atorvastatin and rosuvastatin) when they come off patent in 2012, but much larger price cuts will be needed if Australia (2008A$1.47 per 40 mg simvastatin [37]) is to match prices paid in New Zealand (2008A$0.06 per 40 mg simvastatin [66]) or the United Kingdom (2008A$0.11 per 40 mg simvastatin [67]).

Our results are broadly consistent with the results of previous analyses from WHO-CHOICE [11], Argentina [22] and Vietnam [21]. Our exclusion of aspirin from the optimal intervention package recommended in WHO-CHOICE, and replacement of a beta-blocker with a combination of ACE-inhibitor and calcium channel blocker, better reflect cost-effectiveness based on current drug choices and up-to-date evidence of drug efficacy. The cost-effective (even cost-savings) of a population-wide approach to salt reduction in this modelling study is consistent with the results of all three previous studies that evaluated the relative cost-effectiveness of interventions for primary prevention of CVD. Policy-makers and food manufacturers would do well to heed this growing body of evidence showing the large population health gains to be made by moderating salt use in processed foods.

While cost-effectiveness ratios for the individually-targeted lifestyle interventions were very unfavourable, our analyses do not capture any additional benefits from reduced smoking, increased physical activity, or other possible lifestyle changes, unlike with the analyses of drug interventions, where health benefits are entirely mediated by changes in modelled blood pressure or cholesterol. This means that we are likely underestimating the health benefits of the lifestyle interventions. We do find, however, that even if we add in the DALYs and treatment costs averted by lifestyle intervention changes in physical activity, fruit and vegetable intake, weight loss, alcohol intake and smoking, which have all been modelled separately in other comparable Australian analyses [68], the cost-effectiveness ratio for the lifestyle program is still unfavourable (∼$76,000/DALY) despite likely double-counting of cardiovascular disease benefits. A more accurate analysis of the combined DALY effect, taking interactions in lifestyle risks and correlations in risk behaviours in individuals into account, is however recommended.

The policy-makers behind England’s Vascular Check program should be concerned about the potentially poor value for money of the lifestyle behaviour-change interventions in Australia. The Vascular Check program was predicted to be highly cost-effective by England’s Department of Health [20], but their estimate of health gain was based on summing selected QALY values gathered from a range of other intervention studies, rather than modelling epidemiological outcomes of the intervention combinations in the population over time, taking target population characteristics (e.g. age and sex-specific mortality, blood pressure and cholesterol distributions), long-term disease trends and combined intervention effects into account. Assistance with changing lifestyle needs to be an option, particularly for those wanting to avoid medication in the first instance, but England’s Department of Health would be wise to thoroughly evaluate cost-effectiveness of the current pilot programs before rolling the program out on a national scale, to guard against the possibility of major cost blow-outs with only negligible improvements in population health.

It is also important to evaluate the longer term outcomes of the lifestyle and other cardiovascular disease interventions. Trials of lifestyle interventions in particular are often short-term (e.g. less than two years follow-up). We have assumed that the effects (and costs) of these interventions will be sustained for those who continue to participate, but further evidence is needed to clarify the sustainability of different intervention approaches.

In Australia, it is vital that policy-makers recognise just how far away the country is from optimal prevention of CVD. The remedy is three-fold. The first step is to stand firm against industry pressure and redress current policies around statin dug pricing; this alone would produce immediate Government savings of $500 million in the first year. The second step is to address current inefficiencies in primary care. Australian GPs have been slow to adopt tools for absolute risk assessment [69] with prescribing still largely guided by a confusing mix of rules and criteria defining thresholds for treatment of high blood pressure and cholesterol. The various guidelines are currently being unified, which will remove some of the confusion, but it is vital that GPs are given sufficient information, incentives and support to ensure that absolute risk-based screening and prescription of the most cost-effective drug options become standard practice. Web-based tools that integrate cardiovascular absolute risk assessment with electronic medical record systems may also be of benefit [70]. Thirdly, and most importantly, the Australian Government must enforce moderate salt limits in some processed foods. Limits are currently voluntary for food manufacturers. While the industry may initially resist change, the enforcement of limits will lead to large and immediate improvements in population health.

## Supporting Information

Text S1 Cost-effectiveness model.
(DOC)

Text S2 Model input data.
(DOC)

Text S3 Cost-effectiveness sensitivity results.
(DOC)
