# Supplementary material for: Which Interventions Offer Best Value for Money in Primary Prevention of Cardiovascular Disease?
Source: PLoS One. 2012 Jul 23;7(7):e41842. doi: 10.1371/journal.pone.0041842 (PMC3402472; doi:10.1371/journal.pone.0041842)
Supplement: Text S1 — Cost-effectiveness model. (DOC) [file pone.0041842.s001.doc]

# Text S1: Cost-effectiveness model

1. Model structure

The ischaemic heart disease (IHD) and stroke prevention model is a deterministic Markov model [1] that provides information on health and health care costs over the lifetime of the Australian population (or population sub-groups). The model includes four mutually exclusive health states, with transition between states based on age, sex and multivariate risk-adjusted transition probabilities (Figure 1).

Each five-year age and sex cohort initially begins in the *Alive* state before any incident IHD event (hospitalised angina or acute myocardial infarction) or stroke event (cerebral infarction or intracerebral haemorrhage). Over time, proportions of the cohort may move into an *Alive* state following an IHD event or an *Alive* state following a stroke event, with incident events divided into those that are fatal within the first year and those that survive the first year. Survivors of a first-ever event are at increased risk of death from IHD or Stroke, but may also die from other causes. Transitions are simulated annually until all of the cohort population are dead or have reached 100 years of age.

Although we do not explicitly model ischaemic and haemorrhagic sub-types of stroke as different states, differential rates of incidence and case fatality for either type of stroke event that are fatal within the first year, are taken into account in the transition probabilities. Incidence and case fatality for GI bleeds (a side effect of aspirin therapy) are accounted for in those who are receiving aspirin therapy in any state of the model.


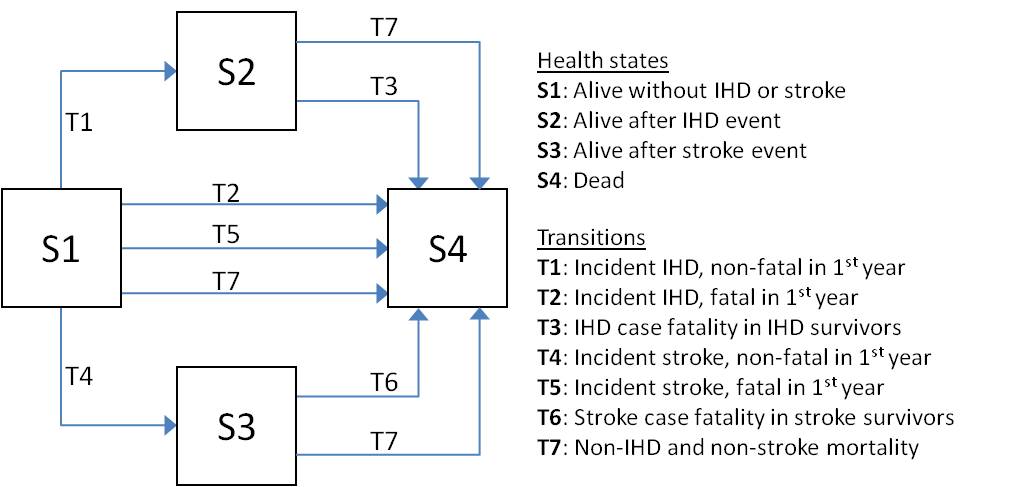


Figure 1 Markov model for simulating IHD and stroke events

1. Transition probabilities
   1. Ischaemic heart disease

The incidence of 28-day survivors of first ever IHD is derived from recorded cases of acute myocardial infarction and unstable angina in the National Hospital Morbidity Database [2]. We then determine the incidence rate in those without previous IHD or stroke, using relative risks of incident IHD in those with stroke form the Busselton study [3,4] and prevalence of stroke in those without previous IHD from AusDiab [5].

Differential rates of case fatality from AMI and angina in the first 28 days are taken into account using in-hospital 28-day case fatality from the National Hospital Morbidity Database [2] and estimates of pre-hospital case fatality from the Perth MONICA study [6]. We calculate case fatality from IHD as the excess mortality in prevalent IHD survivors compared to total population mortality risks using the Western Australia Linked Hospital Database [7,8].

- 1. Stroke

The incidence of 28-day survivors of first-ever stroke is derived from the population-based NEMESIS study [9] and Australian hospital separations data [2]. NEMESIS study incidence is scaled to a national level incidence using the ratio of hospital admissions in the NEMESIS study postcode areas at the time of the NEMESIS study, to hospital admissions Australia-wide in 2003. We adjust the stroke incidence rate to reflect the incidence rate in those without either previous IHD or stroke, using relative risks of incident stroke in those with IHD form the Busselton study [3,4] and prevalence of IHD in those without previous stroke from AusDiab [5].

We derive separate rates of case fatality from ischaemic and haemorrhagic stroke events within the first 28 days from the NEMESIS study [9] and case fatality from stroke in 28-day survivors using the Western Australia Linked Hospital Database [8,10].

- 1. GI bleeds

Incidence of GI bleeds is derived from incidence rates in the National Hospital Morbidity Database [2], excluding cases with a known underlying cause of cancer, cirrhosis/portal hypertension, vascular malformation in the gastrointestinal tract, or inflammatory bowel disease. We derive incidence in those without IHD or stroke, taking account of the prevalence of IHD or stroke in AusDiab [5], the proportion of those with cardiovascular disease taking aspirin in the National Health Survey 2007-08 [11] and the relative risk of a GI bleed in those with IHD or stroke [12].

Case fatality is derived from Victorian Inpatient Minimum Database for 2000, taking the proportion of deaths in hospital in patients admitted for GI bleeds, excluding admissions with a diagnostic code for malignancy or cirrhosis.

- 1. Background mortality

Mortality from all causes other than IHD and stroke is calculated from the total number of deaths recorded by the Australian Bureau of Statistics [13] excluding deaths attributed to IHD or Stroke in the 2003 Australian Burden of Disease study [7], projected to 2008 using projections from Australian mortality data [14].

- 1. Disease trends

Future trends in IHD and stroke incidence and case fatality are based on projections made by Begg et al. [14] from observed Australian mortality between 1979 and 2003. We assume that 58% of the trend is attributable to declining incidence (i.e. due to changes in disease risk factors) and 42% to declining case fatality (i.e. due to changes in survival of those with disease) [15]. The trends are applied for the first 15 years with rates assumed to remain constant thereafter.

1. Disability or quality of life adjustment

At each cycle of the Markov model, we calculate years of life lived within each health state, adjusting for time spent in ill health due to IHD, stroke, a GI bleed and all other causes.

To calculate health gain in DALYs, we derive average Australian disability weights, by age and sex, for IHD, for stroke and for all other causes, from the Australian Burden of Disease study [7]. In addition, all new 28-day IHD survivors receive the Global Burden of Disease [16] disability weight for acute myocardial infarction for the first six weeks of the cycle. The proportion of the population who are alive and experience a GI bleed also receive a one-month disability weight equivalent to the disability associated with an untreated peptic ulcer [17].

To calculate health gain in QALYs, we correct utility weights for angina, congestive heart failure, myocardial infarction and ulcer, from the Beaver dam study [18] using US population norms for background quality of life [19]. For stroke, we correct utility weights derived in the NEMESIS stroke study for background quality of life using AQoL population norms [20]. Average population utility weights are derived from AQoL measures for the Australian population [21].

Table shows the comparison between QALY and DALY weights for the modelled diseases, and Figure A2 shows the comparison for ‘average’ quality of life or ‘background disability’ in Australia. Note that the DALY disability weight measures health lost rather than quality of life experienced and is therefore the complement of the utility weight (disability weight + utility weight = 1).

Table A1 QALY utility weights and DALY disability weights for modelled heart diseases and ulcer/GI bleed (NB. 0 represents the worst possible health state and 1 represents normal health).

| **Disease** | **QALY utility weight** | **1- DALY disability weight** |
| --- | --- | --- |
| Angina | 0.904 [18,19]* | 0.896 [7] |
| Congestive heart failure | 0.863 [18,19]* | 0.809 [7] |
| Myocardial infarction*** | 0.877 [18,19]* | 0.605 [7] |
| Ulcer | 0.918 [18,19]* | 0.936 [7] |
| Stroke | 0.76 [20]** | 0.68 [7] |
| * Beaver dam study utility weights (Quality of Well-being index) corrected for ‘background disability’ using US population norms (Quality of Well-being index) [19], combined multiplicatively, assuming the ‘average’ age for each condition is the same as in Australia [7]  ** NEMESIS stroke study utility weights, corrected for ‘background disability’ using AQoL population quality of life norms [20]  *** QALY utility weights represent utility in those who experienced a myocardial infarction in the last year, while DALY disability weights reflect disability over the three months following myocardial infarction. | | |

Figure A2 Average quality of life or ‘background disability’ for Australians derived using the Assessment of Quality of Life (AQoL) tool for the QALY [21], and the rate of prevalent years of life lived with disability (pYLD), derived from disability weights in the Australian Burden of Disease study [7], for the DALY (NB. 0 represents the worst possible health state and 1 represents normal health).

1. Costs of disease treatment

Costs of treating IHD are determined separately for the first and subsequent years of treatment. First year costs are derived from a Victorian Government study [22] of hospital inpatient costs for IHD treatment and rehabilitation admissions, and from a study by Lim [23] of government and patient out-of-hospital costs for consultations, drugs and diagnostic procedures, and Medicare rebates for private-sector consultations and procedures. Three-quarters of first-year costs are assumed to recur in subsequent years. We derive the direct costs of treating stroke in the first year after an event and annual costs of treatment in subsequent years from the NEMESIS study of stroke costs [24]. Costs of hospital treatment for GI bleeds are from the Victorian Government study [22] of hospital inpatient costs. We adjust all costs to Australian dollars in the year 2008 using health system deflators [25].

1. Uncertainty analysis

Ninety-five percent uncertainty intervals are derived for all cost and health outcome measures by Monte Carlo analysis (2000 iterations) using the Excel Add-In program @Risk (Palisade, Version 4.5). Table A3 shows the uncertainty distributions, sources and assumptions for the model input parameters.

Table A3 Model input parameters and their uncertainty distributions

| **Parameter** | **Value**  Mean (SE) | **Uncertainty distribution** | **Sources and assumptions** |
| --- | --- | --- | --- |
| **Diuretic** |  |  |  |
| Relative risk of disease incidence with diuretic   - IHD - Stroke | 0.86 (0.06)  0.62 (0.05) | Normal (lnRR) | Meta-analyses of primary prevention trials [26,27,28] |
| Annual cost of low-dose diuretic therapy [28] | $69.35 | – | Average annual cost for the standard daily dose [27] of hydrochlorothiazide, chlorthalidone and indapamide, weighted by scripts provided in 2008 [28]. |
| **Beta-blocker** |  |  |  |
| Relative risk of disease incidence with drug treatment beta-blocker   - IHD - Stroke | 0.89 (0.06)  0.83 (0.07) | Normal (lnRR) | Meta-analyses of primary prevention trials [26,27,28] |
| Annual cost of beta-blocker therapy[28] | $105.85 | – | Average annual cost for the standard daily dose [27] of atenolol, metropolol, propranolol, oxprenolol and pindolol, weighted by scripts provided in 2008 [28]. |
| **Calcium channel blocker** |  |  |  |
| Relative risk of disease incidence with calcium channel blocker   - IHD - Stroke | 0.85 (0.04)  0.66 (0.04) | Normal (lnRR) | Meta-analyses of primary prevention trials [26,27,28] |
| Annual cost of calcium channel blocker therapy [28] | $219.00 | – | Average annual cost for the standard daily dose [27] of verapimil, amlodopine (maleate), nifedipine, felodipine, amlodopine (besylate) and lercanidipine, weighted by scripts provided in 2008 [28]. |
| **ACE inhibitor** |  |  |  |
| Relative risk of disease incidence with ACE inhibitor   - IHD - Stroke | 0.83 (0.03)  0.78 (0.07) | Normal (lnRR) | Meta-analyses of primary prevention trials [26,27,28] |
| Annual cost of ACE inhibitor therapy [28] | $211.70 | – | Average annual cost for the standard daily dose [27] of captopril, fosinopril, enalopril, ramipril, quinapril, lisinopril, trandolopril and perindopril, weighted by scripts provided in 2008 [28]. |
| **Statin** |  |  |  |
| Relative risk of disease incidence with statin   - IHD - Stroke | 0.70 (0.05)  0.81 (0.06) | Normal (lnRR) | Meta-analyses of primary prevention trials [28,29,30,31,32] |
| Annual cost of statin therapy[28] | $686.20 | – | Average annual cost for the standard daily dose [27] of fluvastatin, simvastatin, atorvastatin, pravastatin and rosuvastatin, weighted by scripts provided in 2008 [28]. |
| Annual cost of statin therapy in New Zealand [31] | $18.25 | – | Average annual cost of simvastatin (40mg/day) in New Zealand. |
| **Aspirin** |  |  |  |
| Relative risk of disease incidence with aspirin   - IHD - Stroke (ischaemic) - Stroke (haem.) - GI bleed | 0.82 (0.04)  0.86 (0.07)  1.32 (0.19)  1.54 (0.13) | Normal (lnRR) | Meta-analyses of primary prevention trials [12,28,33,34] |
| Annual cost of aspirin therapy [28,33,34] | $40.15 | – | Average annual cost, assuming 50% of aspirin purchased by prescription and 50% over-the-counter. |
| Proportion of aspirin purchased over-the-counter | 50% (10%) | Beta | Estimate |
| ***GP visits – all pharmaceuticals*** |  |  |  |
| Long GP visit [35] | $63.75 | – | One visit in Year 1. |
| Short GP visit [35] | $33.55 | – | Two visits in Year 1 (with BP-lowering drugs) and two visits in Year 2+. |
| Blood test (MBS Item 66512) [35] | $17.80 | – | One test in Year 1 (with lipid-lowering drugs) or three tests in Year 1 (with BP-lowering drugs), and two tests in Year 2+. |
| **Phytosterol margarine** |  |  |  |
| Reduction in total cholesterol with phytosterol margarine | 7.5% (1.9%) | Normal | Meta-analysis of plant sterol/stanol studies [36] |
| Annual cost of phytosterol margarine | $258 ($38) | Gamma | Average annual cost for a mean daily dose of 3.4 g of plant sterols [36] across 20 products available from the two major Australian supermarket chains ([www.woolworths.com.au](http://www.woolworths.com.au/); [www.colesonline.com.au](http://www.colesonline.com.au/)), assuming products contain 82 g of plant sterols per kg of margarine (Food Standards Australia New Zealand Code – Standard 2.4.2) |
| **Dietary advice** |  |  |  |
| Reduction in systolic blood pressure and total cholesterol with dietary advice   - Systolic blood pressure - Total cholesterol | 1.6% (0.4%)  3.1% (1.2%) | Normal | Meta-analysis of randomised controlled trials of dietary advice for primary prevention of cardiovascular disease [37]. |
| Number of dietitian visits with dietary advice   - First year initial - First year follow-up - Subsequent years | 0.5 (1)  1 + 0.5 (1)  1 + 0.5 (1) | Gamma | Fifty percent of patients assumed to receive initial advice from GP and 50% receive referral to dietitian. Minimum of one follow-up visit assumed in first year for those referred to a dietitian. Minimum of one dietitian visit assumed in subsequent years (for adherent patients). |
| Cost of dietitian visit   - Initial visit - Subsequent visit | $80.30  $57.55 | – | Items DT01 and DT20 in the Dietitians Schedule of Fees 2008 (www.dva.gov.au/service_providers/Fee_schedules/Pages/Dental_and_Allied_Health.aspx) |
| **Lifestyle program** |  |  |  |
| Reduction in systolic blood pressure and total cholesterol with lifestyle program   - Systolic blood pressure - Total cholesterol | 2.6% (0.5%)  3.3% (0.6%) | Normal | Meta-analysis of multi-risk factor intervention trials in a health care setting (not including drug therapy), identified in the systematic review by Ebrahim et al [38]. |
| Number of dietitian visits with lifestyle program   - First year initial - First year follow-up - Subsequent years | 1  1 + 0.5 (1)  1 + 0.5 (1) | Gamma | Patients assumed to receive referral to a dietitian in the first year, with a minimum of one follow-up visit in the first year and a minimum of one visit in subsequent years (for adherent patients). |
| Cost of dietitian visit   - Initial visit - Subsequent visit | $80.30  $57.55 | – | Items DT01 and DT20 in the Dietitians Schedule of Fees 2008 (www.dva.gov.au/service_providers/Fee_schedules/Pages/Dental_and_Allied_Health.aspx) |
| Number of exercise physiologist visits with lifestyle program   - First year - Subsequent years | 1 + 0.5 (1)  1 + 0.5 (1) | Gamma | Minimum of one visit assumed in the first year and subsequent years (for adherent patients). |
| Cost of exercise physiologist visit   - Individual session - Group session | $80.30  $57.55 | – | Items EP01 and EP07 in the Exercise Physiologists Schedule of Fees 2008 (www.dva.gov.au/service_providers/Fee_schedules/Pages/Dental_and_Allied_Health.aspx) |
| **Community heart health program** |  |  |  |
| Reduction in systolic blood pressure and total cholesterol with community heart health program   - Systolic blood pressure - Total cholesterol | 2.5% (0.7%)  -0.51% (0.6%) | Normal | Meta-analysis of community heart health program cohort studies identified in the systematic review by Pennant et al [39]. |
| Annual per person cost of a community heart health program   - First year - Subsequent years | $2.37 ($0.47)  $1.60 ($0.32) | Gamma | Estimated from ‘start-up phase’ costs and the equivalent annual cost of the ‘intervention phase’ for the Hartslag Limburg cardiovascular prevention project [40]. |
| **Mandatory salt reduction** |  |  |  |
| Daily per person reduction in sodium intake with mandatory salt reduction   - Men - Women | 10.6 (0.74)  7.3 (0.53) | Normal | Derived from a New Zealand study of sodium reduction in breads, margarines and cereals [41], adjusted for Australian consumption [42]. Methods described in Cobiac et al. [43]. |
| Annual per person cost of mandatory salt reduction program | $0.49 ($0.05) | Gamma | Costs of legislative changes and enforcement for mandatory program derived from resource use[44] and World Health Organisation unit costs ([www.who.int/choice/costs/en/](http://www.who.int/choice/costs/en/)). Standard error assumed to 10% of point estimate. |
| **Voluntary salt reduction** |  |  |  |
| Daily per person reduction in sodium intake with current voluntary salt reduction program   - Men - Women | 0.50 (0.03)  0.34 (0.02) | Normal | Change in mmol/day derived from a New Zealand study of sodium reduction in breads, margarines and cereals [41], adjusted for Australian consumption [42]. Methods described in Cobiac et al. [43]. |
| Annual per person cost of current voluntary salt reduction program | $0.81 ($0.08) | Gamma | Voluntary program cost derived from annual per product fee for participating in Heart Foundation Tick program (C. Colyer, Heart Foundation; personal communication, 18 June 2009). Standard error assumed to 10% of point estimate. |
| **Other modelling parameters** |  |  |  |
| Change in disease incidence with 1% change in total cholesterol   - IHD - Stroke | 1.8% (0.23%)  0.80% (0.20%) | Normal | Estimate from meta-analyses of randomised controlled trials of statin drugs [45,46]. |
| Change in disease incidence with 1% change in systolic blood pressure   - IHD - Stroke | 3.4% (0.39%)  6.3% (0.59%) | Normal | Estimate from meta-analysis of randomised controlled trials of blood pressure-lowering drugs [26]. |
| Change in systolic blood pressure (mmHg) for each 100mmol/24h  change in sodium intake  Mean:   - 30-39 - 40-49 - 50-59 - 60-69   Standard deviation:   - 30-39 - 40-49 - 50-59 - 60-69 | 5.5 (0.9)  6.6 (1)  9.2 (1.2)  10.3 (1.3)  2.0 (0.5)  2.6 (0.6)  3.3 (0.7)  2.9 (0.9) | Normal | Derived from the regressions models developed by Law et al. [47] |
| First year discontinuation with individually-targeted interventions | 40% (8%) | Beta | Estimate from Australian data on discontinuation of statin and blood pressure-lowering drugs [48,49]. Standard error assumed to be 20% of point estimate. |
| RR IHD in stroke   - Men - Women | 2.64 (0.07)  2.85 (0.04) | Normal (lnRR) | Busselton study [3,4] |
| RR GI bleed in IHD/Stroke | 2.69 (0.39) | Normal (lnRR) | Meta-analysis of secondary prevention trials [12] |
| Proportion of IHD/Stroke patients taking aspirin | 24.9% (4%) | Beta | National Health Survey 2007-08 [11] |
| Proportion of GPs participating in primary prevention program | 65% (6.5%) | Beta | Estimate from GP and GP practice participation in the Practice Incentives Program scheme [50]. |
| **Treatment costs** |  |  |  |
| IHD   - First year - Subsequent years | $12,921  $4,539 | Uniform | Lim [23]. Uniform distribution assumed to vary by ±25% around mean. |
| Stroke   - First year - Subsequent years | $23,581  $3,201 | Uniform | Lim [23]. Uniform distribution assumed to vary by ±25% around mean. |
| GI bleed   - Men - Women | $912  $769 | Uniform | Victorian Government study [22]. Uniform distribution assumed to vary by ±25% around mean. |
| NB. All costs adjusted to 2008 Australian dollars using Australian health price deflators [25], consumer price index [51] and/or purchasing power parities [52] where relevant. | | | |

References

1. Sonnenberg F, Beck J (1993) Markov Models in Medical Decision Making: A Practical Guide Medical Decision Making 13: 322-338.

2. AIHW National hospital morbidity database Australian Institute of Health and Welfare.

3. Knuiman MW, Vu HT, Bartholomew HC (1998) Multivariate risk estimation for coronary heart disease: the Busselton Health Study. Australian and New Zealand Journal of Public Health 22: 747-753.

4. Knuiman MW, Vu HTV (1996) Risk factors for stroke mortality in men and women: the Busselton Study. European Journal of Cardiovascular Prevention and Rehabilitation 3: 447-452.

5. Dunstan D, Zimmet P, Welborn T, Sicree R, Armstrong T, et al. (2001) Diabesity and associated disorders in Australia - 2000, The Australian Diabetes, Obesity and Lifestyle Study (AusDiab). Melbourne: International Diabetes Institute.

6. McElduff P, Dobson A, Jamrozik K, Hobbs M (2000) The WHO MONICA Study, Australia, 1984-93: A summary of the Newcastle and Perth MONICA projects. Canberra: Australian Institute of Health and Welfare.

7. Begg S, Vos T, Barker B, Stanley L, Lopez A (2008) Burden of disease and injury in Australia in the new millennium: measuring health loss from diseases, injuries and risk factors. Medical Journal of Australia 188: 36-40.

8. Department of Health Western Australian Data Linkage. Department of Health (Western Australia), The University of Western Australia, Curtin University of Technolgoy, Telethon Institute of Child Health Research.

9. Thrift A, Dewey H, Macdonell R, McNeil J, Donnan G (2000) Stroke Incidence on the east coast of Australia : The North East Melbourne Stroke Incidence Study (NEMESIS). Stroke 31: 2087-2092.

10. Katzenellenbogen JM, Vos T, Somerford P, Begg S, Semmens JB, et al. (2010) Excess Mortality Rates for Estimating the Non-Fatal Burden of Stroke in Western Australia: A Data Linkage Study. Cerebrovascular Diseases 30: 57-64.

11. ABS (2009) National Health Survey: Summary of results, 2007-2008 (Reissue). Canberra: Australian Bureau of Statistics.

12. Antithrombotic Trialists Collaboration (2009) Aspirin in the primary and secondary prevention of vascular disease: collaborative meta-analysis of individual participant data from randomised trials. The Lancet 373: 1849-1860.

13. ABS (2009) Deaths, Australia, 2008. Canberra: Australian Bureau of Statistics.

14. Begg S, Vos T, Goss J, Mann N (2008) An alternative approach to projecting health expenditure in Australia. Australian Health Review 32: 148-155.

15. Unal B, Critchley JA, Capewell S (2004) Explaining the Decline in Coronary Heart Disease Mortality in England and Wales Between 1981 and 2000. Circulation 109: 1101-1107.

16. Murray CJL, Lopez AD (1997) Global mortality, disability, and the contribution of risk factors: Global Burden of Disease Study. The Lancet 349: 1436-1442.

17. Stouthard M, Essink-Bot M, Bonsel G, Barendregt J, Kramers P, et al. (1997) Disability weights for diseases in the Netherlands. the Netherlands: Department of Public Health, Erasmus University Rotterdam.

18. Fryback DG, Dasbach EJ, Klein R, Klein BEK, Dorn N, et al. (1993) The Beaver Dam Health outcomes study: Initial catalog of health state quality factors. Medical Decision Making 13: 89-102.

19. Hanmer J, Lawrence WF, Anderson JP, Kaplan RM, Fryback DG (2006) Report of nationally representative values for the noninstitutionalized US adult population for 7 health-related quality-of-life scores. Medical Decision Making 26: 391-400.

20. Cadilhac DA, Dewey HM, Vos T, Carter R, Thrift AG (2010) The health loss from ischemic stroke and intracerebral hemorrhage: evidence from the North East Melbourne Stroke Incidence Study (NEMESIS). Health and Quality of Life Outcomes 8.

21. Hawthorne G, Osborne R (2005) Population norms and meaningful differences for the Assessment of Quality of Life (AQoL) measure. Australian and New Zealand Journal of Public Health 29: 136-142.

22. KPMG Health Education and Community Services Group (2002) Cost weight study. Melbourne, Victoria: Department of Human Services.

23. Lim S (2005) Priority setting for the primary prevention of coronary heart disease and stroke in Australia [PhD thesis]. Melbourne: Monash University.

24. Dewey HM, Thrift AG, Mihalopoulos C, Carter R, Macdonell RAL, et al. (2001) Cost of stroke in Australia from a societal perspective: results from the North East Melbourne Stroke Incidence Study (NEMESIS). Stroke 32: 2409-2416.

25. AIHW (2006) Health expenditure Australia. Canberra: Australian Institute of Health and Welfare.

26. Law MR, Morris JK, Wald NJ (2009) Use of blood pressure lowering drugs in the prevention of cardiovascular disease: meta-analysis of 147 randomised trials in the context of expectations from prospective epidemiological studies. British Medical Journal 338: b1665.

27. Law MR, Wald NJ, Morris JK, Jordan RE (2003) Value of low dose combination treatment with blood pressure lowering drugs: analysis of 354 randomised trials. BMJ 326: 1427.

28. PBS (2008) Pharmaceutical Benefits Schedule. Canberra: Department of Health and Ageing, Commonwealth of Australia.

29. AIHW (2009) Health expenditure Australia 2007-08. Canberra: Australian Institute of Health and Welfare.

30. Brugts JJ, Yetgin T, Hoeks SE, Gotto AM, Shepherd J, et al. (2009) The benefits of statins in people without established cardiovascular disease but with cardiovascular risk factors: meta-analysis of randomised controlled trials. BMJ 338: b2376-.

31. PHARMAC (2010) Pharmaceutical Schedule. New Zealand: Pharmaceutical Management Agency.

32. Weng TC, Yang YHK, Lin SJ, Tai SH (2010) A systematic review and meta-analysis on the therapeutic equivalence of statins. Journal of Clinical Pharmacy and Therapeutics 35: 139-151.

33. MIMS Online (2010).

34. Pharmacy Direct (2010).

35. MBS (2008) Medicare Benefits Schedule. Canberra: Department of Health and Ageing, Commonwealth of Australia.

36. Chen JT, Wesley R, Shamburek RD, Pucino F, Csako G (2005) Meta-analysis of natural therapies for hyperlipidemia: Plant sterols and stanols versus policosanol. Pharmacotherapy 25: 171-183.

37. Brunner E, Rees K, Ward K, Burke M, Thorogood M (2007) Dietary advice for reducing cardiovascular risk. Cochrane Database of Systematic Reviews Issue 4.

38. Ebrahim S, Beswick A, Burke M, Davey Smith G (2006) Multiple risk factor interventions for primary prevention of coronary heart disease. Cochrane Database of Systematic Reviews Issue 4.

39. Pennant M, Davenport C, Bayliss S, Greenheld W, Marshall T, et al. (2010) Community programs for the prevention of cardiovascular disease: a systematic review Am J Epidemiol 172: 501-516.

40. Ronckers ET, Groot W, Steenbakkers M, Ruland E, Ament A (2006) Costs of the 'Hartslag Limburg' community heart health intervention. BMC Public Health 6.

41. Young L, Swinburn B (2002) Impact of the Pick the Tick food information programme on the salt content of food in New Zealand. Health Promotion International 17: 13-19.

42. ABS (1995) National Nutrition Survey: Foods Eaten, Australia, 1995. Canberra: Australian Bureau of Statistics.

43. Cobiac LJ, Vos T, Veerman JL (2010) Cost-effectiveness of interventions to reduce dietary salt intake. Heart 96: 1920-1925.

44. Asaria P, Chisholm D, Mathers C, Ezzati M, Beaglehole R (2007) Chronic disease prevention: health effects and financial costs of strategies to reduce salt intake and control tobacco use. Lancet 370: 2044-2053.

45. De Caterina R, Scarano M, Marfisi R, Lucisano G, Palma F, et al. (2010) Cholesterol-Lowering Interventions and Stroke: Insights From a Meta-Analysis of Randomized Controlled Trials. Journal of the American College of Cardiology 55: 198-211.

46. Law MR, Wald NJ, Thompson SG (1994) By how much and how quickly does reduction in serum cholesterol concentration lower risk of ischaemic heart disease? British Medical Journal 308: 367-373.

47. Law MR, Frost CD, Wald NJ (1991) By how much does dietary salt reduction lower blood-pressure? 1. Analysis of observational data among populations. British Medical Journal 302: 811-815.

48. Simons L, Simons J, McManus P, Dudley J (2000) Discontinuation rates for use of statins are high [Letter]. BMJ 321: 1084.

49. Simons LA, Ortiz M, Calcino G (2008) Persistence with anti hypertensive medication: Australia-wide experience, 2004-2006. Medical Journal of Australia 188: 224-227.

50. Department of Health and Ageing (2005) General Practice in Australia: 2004. Canberra: Commonwealth of Australia.

51. ABS (2006) Consumer Price Index, Australia, June quarter. Canberra: Australian Bureau of Statistics.

52. OECD (2006) Purchasing Power Parities (PPPs) for OECD Countries 1980-2006. Organisation for Economic Co-operation and Development.
