# Supplementary material for: Which Interventions Offer Best Value for Money in Primary Prevention of Cardiovascular Disease?
Source: PLoS One. 2012 Jul 23;7(7):e41842. doi: 10.1371/journal.pone.0041842 (PMC3402472; doi:10.1371/journal.pone.0041842)
Supplement: Text S2 — Model input data. (DOC) [file pone.0041842.s002.doc]

# Text S2: Model input data

**Table 1 Numbers of Australians without ischaemic heart disease or stroke categorised according to the absolute risk of a cardiovascular event in the next five years.**

Table 2 Numbers of Australians receiving interventions for primary prevention of cardiovascular disease.

Table 3 Incidence

Table 4 Case fatality

Table 5 Disability weights

Table 6 Treatment costs

Table 1 Numbers of Australians without ischaemic heart disease or stroke categorised according to the absolute risk of a cardiovascular event in the next five years.

|  | **<5%** | **5% - 9%** | **10% - 14%** | **≥15%** |
| --- | --- | --- | --- | --- |
| Men |  |  |  |  |
| 35-39 | 371,429 | – | – | – |
| 40-44 | 347,480 | 1,992 | – | – |
| 45-49 | 377,911 | 15,600 | 1,422 | – |
| 50-54 | 278,121 | 55,700 | 9,581 | – |
| 55-59 | 181,406 | 102,363 | 17,498 | 422 |
| 60-64 | 82,833 | 106,349 | 39,544 | 16,004 |
| 65-69 | 20,534 | 97,645 | 57,915 | 18,011 |
| 70-74 | 96 | 36,018 | 61,665 | 39,957 |
| 75-79 | – | 3,309 | 24,633 | 85,366 |
| 80+ | – | – | 10,375 | 60,203 |
| Women |  |  |  |  |
| 35-39 | 453,862 | – | – | – |
| 40-44 | 433,062 | 564 | – | – |
| 45-49 | 433,994 | 5,850 | – | – |
| 50-54 | 381,865 | 11,873 | 710 | – |
| 55-59 | 328,260 | 27,988 | 3,422 | 504 |
| 60-64 | 218,806 | 65,250 | 17,239 | 90 |
| 65-69 | 94,299 | 82,587 | 17,028 | 13,747 |
| 70-74 | 33,188 | 78,807 | 37,594 | 17,926 |
| 75-79 | 6,156 | 48,416 | 33,632 | 43,326 |
| 80+ | – | 4,741 | 34,125 | 63,981 |
|  | | | | |

Table 2 Numbers of Australians receiving interventions for primary prevention of cardiovascular disease.

|  | **Salt only** | **Salt+BP** | **Salt+Lip** | **Salt+Diet** | **Salt+Lip+Diet** | **Salt+BP+Lip** | **Salt+BP+Diet** | **Salt+BP+Lip**  **+Diet** |
| --- | --- | --- | --- | --- | --- | --- | --- | --- |
| Men |  |  |  |  |  |  |  |  |
| 35-39 | 364,669 | 5,163 | 1,189 | – | 446 | – | – | – |
| 40-44 | 328,190 | 5,801 | 3,879 | 2,691 | 1,083 | 6,605 | 245 | 1,013 |
| 45-49 | 359,665 | 20,023 | 11,493 | 790 | 118 | 2,054 | 750 | – |
| 50-54 | 279,117 | 32,314 | 19,643 | 309 | 1,065 | 8,791 | 1,545 | 618 |
| 55-59 | 231,426 | 37,530 | 18,071 | 664 | 362 | 13,636 | – | – |
| 60-64 | 175,283 | 34,724 | 22,317 | 881 | 1,811 | 7,243 | 856 | 1,591 |
| 65-69 | 134,754 | 39,400 | 6,017 | 97 | 1,669 | 8,598 | – | 3,552 |
| 70-74 | 72,477 | 36,610 | 7,520 | 1,598 | 537 | 14,214 | 3,333 | 1,460 |
| 75-79 | 79,021 | 24,826 | 3,184 | – | 34 | 2,459 | 3,796 | – |
| 80+ | 49,461 | 12,168 | – | – | – | 8,907 | – | 42 |
| Women |  |  |  |  |  |  |  |  |
| 35-39 | 441,063 | 12,481 | 318 | – | – | – | – | – |
| 40-44 | 415,413 | 13,659 | 3,599 | – | – | 954 | – | – |
| 45-49 | 398,191 | 20,013 | 11,348 | 1,232 | 748 | 7,609 | – | 660 |
| 50-54 | 311,062 | 51,199 | 15,107 | 986 | – | 9,585 | 5,009 | 1,459 |
| 55-59 | 288,564 | 44,522 | 17,434 | – | 144 | 8,645 | 108 | 756 |
| 60-64 | 184,177 | 66,878 | 32,670 | 151 | – | 10,790 | 3,496 | 3,225 |
| 65-69 | 114,919 | 50,212 | 17,215 | 166 | 1,433 | 21,784 | 519 | 1,391 |
| 70-74 | 87,552 | 57,916 | 8,125 | – | 1,809 | 5,177 | 2,195 | 4,758 |
| 75-79 | 57,584 | 46,522 | 15,941 | – | – | 10,825 | 303 | 342 |
| 80+ | 55,764 | 35,832 | 843 | – | 6,233 | 1,810 | 2,365 | – |
| NB. Salt – voluntary salt reduction program; BP – blood pressure-lowering drugs; Lip – lipid-lowering drugs; Diet – Dietary advice | | | | | | | | |

Table 3 Incidence data

| **Age and sex** | **Current practice scenario** | | **‘Do nothing’ scenario** | | **Gastrointestinal bleed** |
| --- | --- | --- | --- | --- | --- |
|  | **IHD 28-day survivors** | **Stroke 28-day survivors** | **IHD 28-day survivors** | **Stroke 28-day survivors** |  |
| Men |  |  |  |  |  |
| 35-39 | 0.001 | 0.000 | 0.001 | 0 | 0.000 |
| 40-44 | 0.002 | 0.000 | 0.002 | 0 | 0.000 |
| 45-49 | 0.003 | 0.001 | 0.004 | 0.001 | 0.000 |
| 50-54 | 0.005 | 0.001 | 0.007 | 0.001 | 0.000 |
| 55-59 | 0.006 | 0.001 | 0.008 | 0.001 | 0.000 |
| 60-64 | 0.009 | 0.001 | 0.013 | 0.001 | 0.001 |
| 65-69 | 0.011 | 0.003 | 0.016 | 0.004 | 0.001 |
| 70-74 | 0.012 | 0.003 | 0.019 | 0.004 | 0.001 |
| 75-79 | 0.013 | 0.007 | 0.019 | 0.01 | 0.001 |
| 80-84 | 0.015 | 0.007 | 0.023 | 0.009 | 0.002 |
| 85-89 | 0.016 | 0.015 | 0.023 | 0.021 | 0.002 |
| 90-94 | 0.017 | 0.015 | 0.025 | 0.021 | 0.002 |
| 95-99 | 0.018 | 0.015 | 0.026 | 0.021 | 0.002 |
| 100+ | 0.018 | 0.015 | 0.026 | 0.021 | 0.002 |
| Women |  |  |  |  |  |
| 35-39 | 0.000 | 0.000 | 0 | 0 | 0.000 |
| 40-44 | 0.001 | 0.000 | 0.001 | 0 | 0.000 |
| 45-49 | 0.001 | 0.001 | 0.001 | 0.001 | 0.000 |
| 50-54 | 0.002 | 0.001 | 0.002 | 0.001 | 0.000 |
| 55-59 | 0.003 | 0.001 | 0.003 | 0.001 | 0.001 |
| 60-64 | 0.004 | 0.001 | 0.004 | 0.001 | 0.001 |
| 65-69 | 0.006 | 0.002 | 0.007 | 0.002 | 0.001 |
| 70-74 | 0.007 | 0.002 | 0.008 | 0.002 | 0.001 |
| 75-79 | 0.009 | 0.005 | 0.01 | 0.006 | 0.002 |
| 80-84 | 0.011 | 0.005 | 0.013 | 0.006 | 0.001 |
| 85-89 | 0.012 | 0.016 | 0.013 | 0.02 | 0.002 |
| 90-94 | 0.013 | 0.016 | 0.015 | 0.02 | 0.002 |
| 95-99 | 0.013 | 0.016 | 0.015 | 0.02 | 0.002 |
| 100+ | 0.014 | 0.016 | 0.016 | 0.02 | 0.002 |
| NB. IHD (ischaemic heart disease) and stroke rates under a ‘do nothing’ or no intervention comparator scenario are back-calculated from current rates, using the current mix of primary preventive interventions in Australia and published evidence of their effectiveness. | | | | | |

Table 4 Case fatality data

|  | **Ischaemic heart disease** | | **Stroke** | | | **Gastrointestinal bleed** |
| --- | --- | --- | --- | --- | --- | --- |
|  | 28-day | Post 28-day | 28-day  (ischaemic) | 28-day  (haemorrhagic) | Post 28-day |  |
| **Men** | | | | | | |
| 35-39 | 0.163 | 0.007 | 0.12 | 0.45 | 0.028 | 0.005 |
| 40-44 | 0.146 | 0.007 | 0.12 | 0.45 | 0.028 | 0.006 |
| 45-49 | 0.146 | 0.007 | 0.12 | 0.45 | 0.028 | 0.009 |
| 50-54 | 0.145 | 0.004 | 0.12 | 0.45 | 0.028 | 0.012 |
| 55-59 | 0.151 | 0.005 | 0.12 | 0.45 | 0.028 | 0.016 |
| 60-64 | 0.160 | 0.010 | 0.12 | 0.45 | 0.028 | 0.023 |
| 65-69 | 0.186 | 0.010 | 0.12 | 0.45 | 0.053 | 0.031 |
| 70-74 | 0.220 | 0.012 | 0.12 | 0.45 | 0.056 | 0.044 |
| 75-79 | 0.281 | 0.018 | 0.12 | 0.45 | 0.088 | 0.060 |
| 80-84 | 0.452 | 0.020 | 0.12 | 0.45 | 0.081 | 0.083 |
| 85-89 | 0.452 | 0.024 | 0.12 | 0.45 | 0.11 | 0.101 |
| 90-94 | 0.452 | 0.032 | 0.12 | 0.45 | 0.11 | 0.101 |
| 95-99 | 0.452 | 0.065 | 0.12 | 0.45 | 0.11 | 0.101 |
| 100+ | 0.452 | 0.112 | 0.12 | 0.45 | 0.11 | 0.101 |
| **Women** | | | | | | |
| 35-39 | 0.113 | 0.008 | 0.12 | 0.45 | 0.02 | 0.005 |
| 40-44 | 0.111 | 0.008 | 0.12 | 0.45 | 0.02 | 0.006 |
| 45-49 | 0.112 | 0.008 | 0.12 | 0.45 | 0.02 | 0.009 |
| 50-54 | 0.109 | 0.009 | 0.12 | 0.45 | 0.02 | 0.012 |
| 55-59 | 0.118 | 0.007 | 0.12 | 0.45 | 0.02 | 0.016 |
| 60-64 | 0.138 | 0.009 | 0.12 | 0.45 | 0.02 | 0.023 |
| 65-69 | 0.159 | 0.013 | 0.12 | 0.45 | 0.048 | 0.031 |
| 70-74 | 0.198 | 0.019 | 0.12 | 0.45 | 0.055 | 0.044 |
| 75-79 | 0.256 | 0.021 | 0.12 | 0.45 | 0.079 | 0.060 |
| 80-84 | 0.405 | 0.032 | 0.12 | 0.45 | 0.098 | 0.083 |
| 85-89 | 0.405 | 0.036 | 0.12 | 0.45 | 0.118 | 0.101 |
| 90-94 | 0.405 | 0.024 | 0.12 | 0.45 | 0.118 | 0.101 |
| 95-99 | 0.405 | 0.050 | 0.12 | 0.45 | 0.118 | 0.101 |
| 100+ | 0.405 | 0.107 | 0.12 | 0.45 | 0.118 | 0.101 |
| NB. Post 28-day case fatality, for the 28-day survivors of a first ever event, reflects the excess risk of dying from all causes, including IHD following a first-ever stroke event, and stroke following a first-ever IHD event. | | | | | | |

Table 5 Disability weights

|  | **Ischaemic heart disease** | | **Stroke** | **Gastrointestinal bleed** | **Background disability** |
| --- | --- | --- | --- | --- | --- |
|  | First 6 weeks | Post 6 weeks |  |  |  |
| **Men** | | | | | |
| 35-39 | 0.395 | 0.047 | 0.328 | 0.064 | 0.062 |
| 40-44 | 0.395 | 0.047 | 0.328 | 0.064 | 0.064 |
| 45-49 | 0.395 | 0.047 | 0.328 | 0.064 | 0.078 |
| 50-54 | 0.395 | 0.047 | 0.328 | 0.064 | 0.087 |
| 55-59 | 0.395 | 0.047 | 0.328 | 0.064 | 0.097 |
| 60-64 | 0.395 | 0.047 | 0.328 | 0.064 | 0.119 |
| 65-69 | 0.395 | 0.047 | 0.369 | 0.064 | 0.153 |
| 70-74 | 0.395 | 0.047 | 0.369 | 0.064 | 0.191 |
| 75-79 | 0.395 | 0.062 | 0.272 | 0.064 | 0.240 |
| 80-84 | 0.395 | 0.077 | 0.272 | 0.064 | 0.286 |
| 85-89 | 0.395 | 0.103 | 0.272 | 0.064 | 0.341 |
| 90-94 | 0.395 | 0.152 | 0.272 | 0.064 | 0.368 |
| 95-99 | 0.395 | 0.152 | 0.272 | 0.064 | 0.387 |
| 100+ | 0.395 | 0.152 | 0.272 | 0.064 | 0.374 |
| **Women** | | | | | |
| 35-39 | 0.395 | 0.061 | 0.201 | 0.064 | 0.063 |
| 40-44 | 0.395 | 0.061 | 0.201 | 0.064 | 0.066 |
| 45-49 | 0.395 | 0.061 | 0.201 | 0.064 | 0.076 |
| 50-54 | 0.395 | 0.061 | 0.201 | 0.064 | 0.082 |
| 55-59 | 0.395 | 0.061 | 0.201 | 0.064 | 0.097 |
| 60-64 | 0.395 | 0.061 | 0.201 | 0.064 | 0.107 |
| 65-69 | 0.395 | 0.093 | 0.272 | 0.064 | 0.115 |
| 70-74 | 0.395 | 0.101 | 0.272 | 0.064 | 0.138 |
| 75-79 | 0.395 | 0.112 | 0.393 | 0.064 | 0.183 |
| 80-84 | 0.395 | 0.128 | 0.393 | 0.064 | 0.233 |
| 85-89 | 0.395 | 0.156 | 0.393 | 0.064 | 0.290 |
| 90-94 | 0.395 | 0.209 | 0.393 | 0.064 | 0.331 |
| 95-99 | 0.395 | 0.209 | 0.393 | 0.064 | 0.348 |
| 100+ | 0.395 | 0.209 | 0.393 | 0.064 | 0.340 |

Table 6 Treatment costs

|  | **Ischaemic heart disease** | | **Stroke** | | **Gastrointestinal bleed** |
| --- | --- | --- | --- | --- | --- |
|  | First year | Subsequent years | First year | Subsequent years |  |
| **Men** | | | | | |
| 35-39 | $12,921 | $4,539 | $23,581 | $3,201 | $430 |
| 40-44 | $12,921 | $4,539 | $23,581 | $3,201 | $381 |
| 45-49 | $12,921 | $4,539 | $23,581 | $3,201 | $483 |
| 50-54 | $12,921 | $4,539 | $23,581 | $3,201 | $455 |
| 55-59 | $12,921 | $4,539 | $23,581 | $3,201 | $543 |
| 60-64 | $12,921 | $4,539 | $23,581 | $3,201 | $660 |
| 65-69 | $12,921 | $4,539 | $23,581 | $3,201 | $1,116 |
| 70-74 | $12,921 | $4,539 | $23,581 | $3,201 | $1,018 |
| 75-79 | $12,921 | $4,539 | $23,581 | $3,201 | $1,350 |
| 80-84 | $12,921 | $4,539 | $23,581 | $3,201 | $1,627 |
| 85-89 | $12,921 | $4,539 | $23,581 | $3,201 | $2,114 |
| 90-94 | $12,921 | $4,539 | $23,581 | $3,201 | $1,935 |
| 95-99 | $12,921 | $4,539 | $23,581 | $3,201 | $1,126 |
| 100+ | $12,921 | $4,539 | $23,581 | $3,201 | $1,126 |
| **Women** | | | | | |
| 35-39 | $12,921 | $4,539 | $23,581 | $3,201 | $394 |
| 40-44 | $12,921 | $4,539 | $23,581 | $3,201 | $267 |
| 45-49 | $12,921 | $4,539 | $23,581 | $3,201 | $340 |
| 50-54 | $12,921 | $4,539 | $23,581 | $3,201 | $303 |
| 55-59 | $12,921 | $4,539 | $23,581 | $3,201 | $320 |
| 60-64 | $12,921 | $4,539 | $23,581 | $3,201 | $454 |
| 65-69 | $12,921 | $4,539 | $23,581 | $3,201 | $622 |
| 70-74 | $12,921 | $4,539 | $23,581 | $3,201 | $837 |
| 75-79 | $12,921 | $4,539 | $23,581 | $3,201 | $1,143 |
| 80-84 | $12,921 | $4,539 | $23,581 | $3,201 | $1,432 |
| 85-89 | $12,921 | $4,539 | $23,581 | $3,201 | $1,602 |
| 90-94 | $12,921 | $4,539 | $23,581 | $3,201 | $1,816 |
| 95-99 | $12,921 | $4,539 | $23,581 | $3,201 | $1,248 |
| 100+ | $12,921 | $4,539 | $23,581 | $3,201 | $1,248 |
