# Supplementary material for: Which Interventions Offer Best Value for Money in Primary Prevention of Cardiovascular Disease?
Source: PLoS One. 2012 Jul 23;7(7):e41842. doi: 10.1371/journal.pone.0041842 (PMC3402472; doi:10.1371/journal.pone.0041842)
Supplement: Text S3 — Cost-effectiveness sensitivity results. (DOC) [file pone.0041842.s003.doc]

# Text S3: Cost-effectiveness sensitivity results

**Table 1** Health gain, costs and cost-effectiveness of interventions in the most cost-effective pathway.

**Table 2** Health gain, costs and cost-effectiveness of interventions in the most cost-effective pathway, when discounting is reduced from 3% to 0%.

**Table 3** Health gain, costs and cost-effectiveness of interventions in the most cost-effective pathway, when discounting is increased from 3% to 5%.

**Table 4** Health gain, costs and cost-effectiveness of interventions in the most cost-effective pathway, when other (non-cardiovascular) health care costs in added years of life are included in the analysis.

**Table 5** Health gain, costs and cost-effectiveness of interventions in the most cost-effective pathway, when the cost of statins is reduced to the current price in New Zealand.

**Table 6** Health gain, costs and cost-effectiveness of interventions in the most cost-effective pathway, when health gain is measured in QALYs rather than DALYs.

Table 1 Health gain, costs and cost-effectiveness of interventions in the most cost-effective pathway.

| **Intervention and target group** | **Lifetime health gain (DALYs)** | **Intervention cost to government (2008A$million)** | **Disease treatment costs averted (2008A$million)** | **Cost-effectiveness when added to the package ($/DALY)*** |
| --- | --- | --- | --- | --- |
| Mandatory salt limits (all risk levels) | 80,000 (60,000 to 100,000) | $84 ($61 to $110) | -$930 (-$1,300 to -$600) | Dominant** (Dominant to Dominant) |
| Comm. heart program (all risk levels) | 2,600 (1,300 to 4,000) | $170 ($110 to $250) | -$30 (-$52 to -$13) | $55,000 ($25,000 to $120,000) |
| Diuretic (≥15% risk) | 38,000 (22,000 to 58,000) | $380 ($270 to $500) | -$380 (-$630 to -$190) | Dominant** (Dominant to $6,100) |
| Diuretic (10-14% risk) | 39,000 (22,000 to 59,000) | $490 ($350 to $640) | -$400 (-$670 to -$180) | $2,300** (Dominant to $10,000) |
| Diuretic (5-9% risk) | 75,000 (42,000 to 110,000) | $1,200 ($880 to $1,700) | -$780 (-$1,300 to -$340) | $6,200** ($170 to $17,000) |
| Ca channel blocker (≥15% risk) | 28,000 (18,000 to 41,000) | $470 ($340 to $630) | -$300 (-$470 to -$170) | $6,300** ($1,600 to $12,000) |
| Ca channel blocker (10-14% risk) | 29,000 (18,000 to 42,000) | $610 ($430 to $820) | -$320 (-$510 to -$180) | $10,000** ($4,400 to $18,000) |
| Ca channel blocker (5-9% risk) | 56,000 (34,000 to 81,000) | $1,600 ($1,100 to $2,100) | -$640 (-$1,000 to -$350) | $16,000** ($9,600 to $28,000) |
| ACE inhibitor (≥15% risk) | 20,000 (12,000 to 30,000) | $480 ($340 to $640) | -$230 (-$370 to -$120) | $12,000** ($6,400 to $23,000) |
| ACE inhibitor (10-14% risk) | 21,000 (13,000 to 31,000) | $620 ($440 to $830) | -$260 (-$420 to -$140) | $17,000** ($9,700 to $29,000) |
| ACE inhibitor (5-9% risk) | 40,000 (24,000 to 61,000) | $1,600 ($1,100 to $2,100) | -$530 (-$850 to -$290) | $26,000** ($16,000 to $42,000) |
| Statin (≥15% risk) | 25,000 (15,000 to 38,000) | $1,700 ($1,200 to $2,200) | -$300 (-$480 to -$160) | $54,000 ($38,000 to $85,000) |
| Statin (10-14% risk) | 27,000 (16,000 to 40,000) | $2,200 ($1,500 to $2,900) | -$360 (-$590 to -$190) | $68,000 ($48,000 to $100,000) |
| Statin (5-9% risk) | 51,000 (30,000 to 77,000) | $5,500 ($3,800 to $7,400) | -$730 (-$1,200 to -$380) | $93,000 ($66,000 to $140,000) |
| Dietary advice (≥15% risk) | 82 (46 to 130) | $200 ($110 to $450) | -$0.9 (-$1.6 to -$0.5) | $2,300,000 ($1,200,000 to $5,900,000) |
| Dietary advice (10-14% risk) | 86 (48 to 140) | $260 ($140 to $580) | -$1.1 (-$1.9 to -$0.5) | $2,700,000 ($1,500,000 to $7,200,000) |
| Dietary advice (5-9% risk) | 160 (91 to 270) | $630 ($350 to $1,400) | -$2.2 (-$3.7 to -$1.1) | $3,500,000 ($1,900,000 to $9,300,000) |
| Phytosterol (≥15% risk) | 80 (38 to 130) | $570 ($350 to $860) | -$0.9 (-$1.7 to -$0.4) | $7,200,000 ($4,100,000 to $14,000,000) |
| Phytosterol (10-14% risk) | 84 (40 to 140) | $730 ($450 to $1,100) | -$1.1 (-$2.1 to -$0.5) | $8,700,000 ($5,000,000 to $17,000,000) |
| Phytosterol (5-9% risk) | 160 (77 to 270) | $1,800 ($1,100 to $2,700) | -$2.3 (-$4.2 to -$1.0) | $11,000,000 ($6,400,000 to $22,000,000) |
| NB. Values are mean and 95% uncertainty interval for health gain and costs, and median and 95% uncertainty interval for cost-effectiveness ratios. All figures are rounded to two significant figures.  ***** Where the ratio is *Dominant*, the intervention is cost-saving.  ****** Indicates interventions that are included in the optimal package when the cost-effectiveness threshold is $50,000/DALY. | | | | |

Table 2 Health gain, costs and cost-effectiveness of interventions in the most cost-effective pathway, when discounting is reduced from 3% to 0%.

| **Intervention and target group** | **Lifetime health gain (DALYs)** | **Intervention cost to government (2008A$million)** | **Disease treatment costs averted (2008A$million)** | **Cost-effectiveness when added to the package ($/DALY)*** |
| --- | --- | --- | --- | --- |
| Mandatory salt limits (all risk levels) | 180,000 (140,000 to 230,000) | $130 ($97 to $170) | -$1,900 (-$2,700 to -$1,200) | Dominant** (Dominant to Dominant) |
| Comm. heart program (all risk levels) | 6,300 (3,200 to 9,800) | $270 ($170 to $390) | -$63 (-$110 to -$28) | $33,000** ($14,000 to $77,000) |
| Diuretic (≥15% risk) | 56,000 (32,000 to 84,000) | $460 ($320 to $600) | -$510 (-$830 to -$250) | Dominant** (Dominant to $4,400) |
| Diuretic (10-14% risk) | 65,000 (37,000 to 99,000) | $620 ($440 to $820) | -$590 (-$1,000 to -$270) | $550** (Dominant to $7,300) |
| Diuretic (5-9% risk) | 140,000 (79,000 to 210,000) | $1,600 ($1,100 to $2,200) | -$1,300 (-$2,200 to -$560) | $2,400** (Dominant to $10,000) |
| Ca channel blocker (≥15% risk) | 41,000 (26,000 to 60,000) | $590 ($410 to $780) | -$400 (-$630 to -$230) | $4,600** ($510 to $9,900) |
| Ca channel blocker (10-14% risk) | 49,000 (30,000 to 70,000) | $800 ($560 to $1,100) | -$480 (-$770 to -$270) | $6,500** ($1,700 to $13,000) |
| Ca channel blocker (5-9% risk) | 110,000 (65,000 to 150,000) | $2,100 ($1,500 to $2,800) | -$1,100 (-$1,700 to -$580) | $9,900** ($4,300 to $17,000) |
| ACE inhibitor (≥15% risk) | 29,000 (18,000 to 45,000) | $600 ($430 to $800) | -$310 (-$500 to -$170) | $10,000** ($4,500 to $18,000) |
| ACE inhibitor (10-14% risk) | 35,000 (21,000 to 53,000) | $820 ($580 to $1,100) | -$400 (-$650 to -$220) | $12,000** ($5,800 to $21,000) |
| ACE inhibitor (5-9% risk) | 76,000 (46,000 to 110,000) | $2,200 ($1,500 to $2,900) | -$900 (-$1,500 to -$490) | $16,000** ($9,100 to $28,000) |
| Statin (≥15% risk) | 37,000 (22,000 to 56,000) | $2,100 ($1,500 to $2,800) | -$410 (-$670 to -$210) | $45,000** ($31,000 to $71,000) |
| Statin (10-14% risk) | 45,000 (26,000 to 68,000) | $2,800 ($2,000 to $3,800) | -$550 (-$910 to -$290) | $51,000 ($35,000 to $80,000) |
| Statin (5-9% risk) | 98,000 (57,000 to 150,000) | $7,500 ($5,200 to $10,000) | -$1,300 (-$2,100 to -$650) | $63,000 ($44,000 to $99,000) |
| Dietary advice (≥15% risk) | 120 (68 to 200) | $250 ($140 to $560) | -$1.3 (-$2.2 to -$0.7) | $1,900,000 ($1,000,000 to $4,900,000) |
| Dietary advice (10-14% risk) | 150 (82 to 240) | $340 ($190 to $770) | -$1.7 (-$2.9 to -$0.8) | $2,000,000 ($1,100,000 to $5,600,000) |
| Dietary advice (5-9% risk) | 320 (180 to 510) | $890 ($490 to $2,000) | -$3.8 (-$6.5 to -$1.8) | $2,500,000 ($1,400,000 to $6,800,000) |
| Phytosterol (≥15% risk) | 120 (56 to 200) | $720 ($440 to $1,100) | -$1.3 (-$2.3 to -$0.6) | $6,100,000 ($3,500,000 to $12,000,000) |
| Phytosterol (10-14% risk) | 140 (68 to 240) | $980 ($600 to $1,500) | -$1.8 (-$3.2 to -$0.8) | $6,800,000 ($3,900,000 to $13,000,000) |
| Phytosterol (5-9% risk) | 310 (150 to 520) | $2,600 ($1,600 to $3,800) | -$4.1 (-$7.3 to -$1.7) | $8,300,000 ($4,700,000 to $16,000,000) |
| NB. Values are mean and 95% uncertainty interval for health gain and costs, and median and 95% uncertainty interval for cost-effectiveness ratios. All figures are rounded to two significant figures.  ***** Where the ratio is *Dominant*, the intervention is cost-saving.  ****** Indicates interventions that are included in the optimal package when the cost-effectiveness threshold is $50,000/DALY. | | | | |

Table 3 Health gain, costs and cost-effectiveness of interventions in the most cost-effective pathway, when discounting is increased from 3% to 5%.

| **Intervention and target group** | **Lifetime health gain (DALYs)** | **Intervention cost to government (2008A$million)** | **Disease treatment costs averted (2008A$million)** | **Cost-effectiveness when added to the package ($/DALY)*** |
| --- | --- | --- | --- | --- |
| Mandatory salt limits (all risk levels) | 51,000 (38,000 to 64,000) | $66 ($50 to $83) | -$630 (-$890 to -$410) | Dominant** (Dominant to Dominant) |
| Comm. heart program (all risk levels) | 1,600 (-2,500 to 5,900) | $140 ($87 to $200) | -$20 (-$81 to $37) | $75,000 ($9,400 to Dominated?) |
| Diuretic (≥15% risk) | 30,000 (17,000 to 46,000) | $340 ($240 to $440) | -$330 (-$530 to -$170) | $520** (Dominant to $7,400) |
| Diuretic (10-14% risk) | 29,000 (16,000 to 44,000) | $430 ($300 to $560) | -$320 (-$540 to -$150) | $3,900** (Dominant to $13,000) |
| Diuretic (5-9% risk) | 52,000 (29,000 to 78,000) | $1,100 ($770 to $1,400) | -$580 (-$1,000 to -$260) | $9,600** ($2,400 to $23,000) |
| Ca channel blocker (≥15% risk) | 22,000 (14,000 to 32,000) | $420 ($300 to $550) | -$250 (-$390 to -$140) | $7,600** ($2,400 to $14,000) |
| Ca channel blocker (10-14% risk) | 21,000 (13,000 to 31,000) | $530 ($380 to $710) | -$250 (-$400 to -$140) | $13,000** ($6,600 to $22,000) |
| Ca channel blocker (5-9% risk) | 38,000 (23,000 to 56,000) | $1,300 ($950 to $1,800) | -$470 (-$750 to -$260) | $23,000** ($14,000 to $36,000) |
| ACE inhibitor (≥15% risk) | 16,000 (9,500 to 24,000) | $420 ($300 to $560) | -$190 (-$310 to -$100) | $14,000** ($7,800 to $26,000) |
| ACE inhibitor (10-14% risk) | 15,000 (9,300 to 23,000) | $540 ($380 to $710) | -$200 (-$330 to -$110) | $21,000** ($12,000 to $36,000) |
| ACE inhibitor (5-9% risk) | 28,000 (17,000 to 42,000) | $1,300 ($940 to $1,800) | -$390 (-$620 to -$210) | $34,000** ($22,000 to $54,000) |
| Statin (≥15% risk) | 20,000 (12,000 to 30,000) | $1,500 ($1,000 to $1,900) | -$250 (-$400 to -$130) | $61,000 ($43,000 to $95,000) |
| Statin (10-14% risk) | 20,000 (11,000 to 29,000) | $1,900 ($1,300 to $2,500) | -$280 (-$450 to -$140) | $81,000 ($58,000 to $120,000) |
| Statin (5-9% risk) | 35,000 (20,000 to 53,000) | $4,700 ($3,300 to $6,200) | -$530 (-$880 to -$270) | $110,000 ($85,000 to $180,000) |
| Dietary advice (≥15% risk) | 65 (36 to 100) | $180 ($100 to $390) | -$0.8 (-$1.3 to -$0.4) | $2,600,000 ($1,400,000 to $6,500,000) |
| Dietary advice (10-14% risk) | 63 (35 to 100) | $220 ($120 to $490) | -$0.8 (-$1.4 to -$0.4) | $3,200,000 ($1,800,000 to $8,400,000) |
| Dietary advice (5-9% risk) | 110 (61 to 180) | $520 ($290 to $1,200) | -$1.6 (-$2.7 to -$0.8) | $4,300,000 ($2,400,000 to $11,000,000) |
| Phytosterol (≥15% risk) | 63 (30 to 100) | $500 ($310 to $750) | -$0.8 (-$1.4 to -$0.3) | $8,000,000 ($4,600,000 to $16,000,000) |
| Phytosterol (10-14% risk) | 62 (29 to 100) | $620 ($380 to $930) | -$0.9 (-$1.6 to -$0.4) | $10,000,000 ($5,800,000 to $20,000,000) |
| Phytosterol (5-9% risk) | 110 (52 to 180) | $1,500 ($910 to $2,200) | -$1.7 (-$3.1 to -$0.7) | $13,000,000 ($7,700,000 to $27,000,000) |
| NB. Values are mean and 95% uncertainty interval for health gain and costs, and median and 95% uncertainty interval for cost-effectiveness ratios. All figures are rounded to two significant figures.  ***** Where the ratio is *Dominant*, the intervention is cost-saving.  ****** Indicates interventions that are included in the optimal package when the cost-effectiveness threshold is $50,000/DALY. | | | | |

Table 4 Health gain, costs and cost-effectiveness of interventions in the most cost-effective pathway, when other (non-cardiovascular) health care costs in added years of life are included in the analysis.

| **Intervention and target group** | **Lifetime health gain (DALYs)** | **Intervention cost to government (2008A$million)** | **Disease treatment costs averted (2008A$million)** | **Cost-effectiveness when added to the package ($/DALY)*** |
| --- | --- | --- | --- | --- |
| Mandatory salt limits (all risk levels) | 110,000 (86,000 to 150,000) | $84 ($61 to $110) | $140 (-$140 to $430) | $1,900** (Dominant to $4,500) |
| Comm. heart program (all risk levels) | 2,600 (1,300 to 4,000) | $170 ($110 to $250) | $05 (-$07 to $17) | $68,000 ($39,000 to $130,000) |
| Diuretic (≥15% risk) | 37,000 (21,000 to 58,000) | $380 ($270 to $490) | $390 ($200 to $640) | $20,000** ($13,000 to $34,000) |
| Diuretic (10-14% risk) | 39,000 (21,000 to 60,000) | $490 ($350 to $640) | $250 ($110 to $450) | $19,000** ($13,000 to $31,000) |
| Diuretic (5-9% risk) | 74,000 (41,000 to 120,000) | $1,200 ($890 to $1,600) | $350 ($69 to $680) | $21,000** ($15,000 to $34,000) |
| Ca channel blocker (≥15% risk) | 28,000 (17,000 to 41,000) | $470 ($340 to $620) | $280 ($150 to $450) | $27,000** ($19,000 to $39,000) |
| Ca channel blocker (10-14% risk) | 29,000 (17,000 to 43,000) | $620 ($440 to $810) | $160 ($59 to $300) | $27,000** ($20,000 to $39,000) |
| Ca channel blocker (5-9% risk) | 55,000 (33,000 to 82,000) | $1,600 ($1,100 to $2,100) | $200 ($07 to $440) | $32,000** ($24,000 to $46,000) |
| ACE inhibitor (≥15% risk) | 20,000 (12,000 to 30,000) | $480 ($350 to $640) | $190 ($94 to $340) | $34,000** ($24,000 to $51,000) |
| ACE inhibitor (10-14% risk) | 21,000 (12,000 to 31,000) | $620 ($440 to $820) | $92 ($10 to $210) | $34,000** ($25,000 to $50,000) |
| ACE inhibitor (5-9% risk) | 40,000 (24,000 to 59,000) | $1,600 ($1,100 to $2,100) | $84 (-$83 to $290) | $41,000** ($31,000 to $60,000) |
| Statin (≥15% risk) | 25,000 (15,000 to 37,000) | $1,700 ($1,200 to $2,200) | $240 ($110 to $410) | $76,000 ($57,000 to $110,000) |
| Statin (10-14% risk) | 26,000 (16,000 to 39,000) | $2,200 ($1,500 to $2,900) | $95 (-$04 to $230) | $86,000 ($65,000 to $120,000) |
| Statin (5-9% risk) | 51,000 (30,000 to 75,000) | $5,500 ($3,900 to $7,300) | $53 (-$160 to $280) | $100,000 ($83,000 to $150,000) |
| Dietary advice (≥15% risk) | 81 (46 to 130) | $210 ($110 to $450) | $0.8 ($0.4 to $1.4) | $2,300,000 ($1,300,000 to $6,100,000) |
| Dietary advice (10-14% risk) | 85 (48 to 130) | $260 ($140 to $590) | $0.4 ($0.1 to $0.9) | $2,700,000 ($1,600,000 to $7,500,000) |
| Dietary advice (5-9% risk) | 160 (92 to 250) | $630 ($350 to $1,500) | $0.4 (-$0.3 to $1.1) | $3,500,000 ($2,000,000 to $9,800,000) |
| Phytosterol (≥15% risk) | 79 (37 to 130) | $570 ($360 to $850) | $0.7 ($0.3 to $1.4) | $7,300,000 ($4,100,000 to $14,000,000) |
| Phytosterol (10-14% risk) | 84 (39 to 140) | $730 ($450 to $1,100) | $0.3 ($0.0 to $0.8) | $8,700,000 ($4,900,000 to $18,000,000) |
| Phytosterol (5-9% risk) | 160 (75 to 270) | $1,800 ($1,100 to $2,700) | $0.1 (-$0.6 to $0.9) | $11,000,000 ($6,200,000 to $23,000,000) |
| NB. Values are mean and 95% uncertainty interval for health gain and costs, and median and 95% uncertainty interval for cost-effectiveness ratios. All figures are rounded to two significant figures.  ***** Where the ratio is *Dominant*, the intervention is cost-saving.  ****** Indicates interventions that are included in the optimal package when the cost-effectiveness threshold is $50,000/DALY. | | | | |

Table 5 Health gain, costs and cost-effectiveness of interventions in the most cost-effective pathway, when the cost of statins is reduced to the current price in New Zealand.

| **Intervention and target group** | **Lifetime health gain (DALYs)** | **Intervention cost to government (2008A$million)** | **Disease treatment costs averted (2008A$million)** | **Cost-effectiveness when added to the package ($/DALY)*** |
| --- | --- | --- | --- | --- |
| Mandatory salt limits (all risk levels) | 80,000 (60,000 to 100,000) | $84 ($61 to $110) | -$930 (-$1,300 to -$600) | Dominant** (Dominant to Dominant) |
| Comm. heart program (all risk levels) | 2,600 (1,300 to 4,000) | $170 ($110 to $250) | -$29 (-$50 to -$13) | $56,000 ($26,000 to $120,000) |
| Diuretic (≥15% risk) | 32,000 (18,000 to 49,000) | $260 ($190 to $340) | -$330 (-$530 to -$170) | Dominant** (Dominant to $3,000) |
| Diuretic (10-14% risk) | 33,000 (19,000 to 51,000) | $330 ($240 to $440) | -$340 (-$560 to -$160) | $110** (Dominant to $6,400) |
| Diuretic (5-9% risk) | 63,000 (35,000 to 96,000) | $840 ($590 to $1,100) | -$650 (-$1,100 to -$290) | $3,100** (Dominant to $11,000) |
| Ca channel blocker (≥15% risk) | 23,000 (14,000 to 34,000) | $490 ($350 to $660) | -$240 (-$380 to -$140) | $10,000** ($5,100 to $18,000) |
| Ca channel blocker (10-14% risk) | 24,000 (15,000 to 35,000) | $640 ($450 to $860) | -$260 (-$400 to -$150) | $16,000** ($9,000 to $26,000) |
| Ca channel blocker (5-9% risk) | 45,000 (28,000 to 66,000) | $1,600 ($1,100 to $2,200) | -$510 (-$800 to -$280) | $25,000** ($16,000 to $38,000) |
| ACE inhibitor (≥15% risk) | 16,000 (9,400 to 24,000) | $500 ($350 to $660) | -$180 (-$290 to -$99) | $20,000** ($11,000 to $34,000) |
| ACE inhibitor (10-14% risk) | 17,000 (9,900 to 25,000) | $640 ($450 to $860) | -$200 (-$330 to -$110) | $26,000** ($16,000 to $43,000) |
| ACE inhibitor (5-9% risk) | 31,000 (19,000 to 48,000) | $1,600 ($1,100 to $2,200) | -$410 (-$660 to -$220) | $38,000** ($25,000 to $60,000) |
| Statin (≥15% risk) | 40,000 (24,000 to 61,000) | $240 ($170 to $320) | -$450 (-$740 to -$240) | Dominant** (Dominant to Dominant) |
| Statin (10-14% risk) | 42,000 (25,000 to 64,000) | $320 ($230 to $430) | -$540 (-$880 to -$290) | Dominant** (Dominant to Dominant) |
| Statin (5-9% risk) | 83,000 (49,000 to 120,000) | $840 ($590 to $1,100) | -$1,100 (-$1,800 to -$600) | Dominant** (Dominant to $2,300) |
| Dietary advice (≥15% risk) | 82 (46 to 130) | $120 ($45 to $350) | -$0.9 (-$1.6 to -$0.5) | $1,100,000 ($560,000 to $4,500,000) |
| Dietary advice (10-14% risk) | 86 (48 to 140) | $170 ($72 to $480) | -$1.1 (-$1.9 to -$0.5) | $1,600,000 ($840,000 to $5,800,000) |
| Dietary advice (5-9% risk) | 160 (91 to 270) | $450 ($200 to $1,200) | -$2.2 (-$3.7 to -$1.1) | $2,200,000 ($1,200,000 to $7,900,000) |
| Phytosterol (≥15% risk) | 80 (38 to 130) | $500 ($300 to $750) | -$0.9 (-$1.7 to -$0.4) | $6,300,000 ($3,600,000 to $12,000,000) |
| Phytosterol (10-14% risk) | 84 (40 to 140) | $660 ($390 to $990) | -$1.1 (-$2.1 to -$0.5) | $7,800,000 ($4,500,000 to $15,000,000) |
| Phytosterol (5-9% risk) | 160 (77 to 270) | $1,700 ($990 to $2,500) | -$2.3 (-$4.2 to -$1.0) | $10,000,000 ($5,800,000 to $20,000,000) |
| NB. Values are mean and 95% uncertainty interval for health gain and costs, and median and 95% uncertainty interval for cost-effectiveness ratios. All figures are rounded to two significant figures.  ***** Where the ratio is *Dominant*, the intervention is cost-saving.  ****** Indicates interventions that are included in the optimal package when the cost-effectiveness threshold is $50,000/DALY. | | | | |

Table 6 Health gain, costs and cost-effectiveness of interventions in the most cost-effective pathway, when health gain is measured in QALYs rather than DALYs.

| **Intervention and target group** | **Lifetime health gain (DALYs)** | **Intervention cost to government (2008A$million)** | **Disease treatment costs averted (2008A$million)** | **Cost-effectiveness when added to the package ($/DALY)*** |
| --- | --- | --- | --- | --- |
| Mandatory salt limits (all risk levels) | 68,000 (51,000 to 87,000) | $84 ($63 to $110) | -$930 (-$1,300 to -$610) | Dominant** (Dominant to Dominant) |
| Comm. heart program (all risk levels) | 2,200 (-3,700 to 8,400) | $170 ($110 to $250) | -$30 (-$130 to $61) | $67,000 ($5,200 to Dominated?) |
| Diuretic (≥15% risk) | 37,000 (21,000 to 56,000) | $380 ($270 to $500) | -$380 (-$630 to -$190) | Dominant** (Dominant to $6,100) |
| Diuretic (10-14% risk) | 36,000 (20,000 to 54,000) | $490 ($350 to $640) | -$400 (-$670 to -$180) | $2,600** (Dominant to $11,000) |
| Diuretic (5-9% risk) | 64,000 (35,000 to 99,000) | $1,200 ($880 to $1,700) | -$780 (-$1,300 to -$340) | $7,200** ($200 to $21,000) |
| Ca channel blocker (≥15% risk) | 27,000 (17,000 to 39,000) | $470 ($340 to $630) | -$300 (-$470 to -$170) | $6,500** ($1,600 to $13,000) |
| Ca channel blocker (10-14% risk) | 26,000 (16,000 to 38,000) | $610 ($430 to $820) | -$320 (-$510 to -$180) | $11,000** ($4,800 to $20,000) |
| Ca channel blocker (5-9% risk) | 48,000 (30,000 to 71,000) | $1,600 ($1,100 to $2,100) | -$640 (-$1,000 to -$350) | $19,000** ($11,000 to $32,000) |
| ACE inhibitor (≥15% risk) | 19,000 (11,000 to 29,000) | $480 ($340 to $640) | -$230 (-$370 to -$120) | $13,000** ($6,700 to $24,000) |
| ACE inhibitor (10-14% risk) | 19,000 (12,000 to 29,000) | $620 ($440 to $830) | -$260 (-$420 to -$140) | $18,000** ($10,000 to $32,000) |
| ACE inhibitor (5-9% risk) | 36,000 (22,000 to 54,000) | $1,600 ($1,100 to $2,100) | -$530 (-$850 to -$290) | $29,000** ($18,000 to $47,000) |
| Statin (≥15% risk) | 24,000 (14,000 to 36,000) | $1,700 ($1,200 to $2,200) | -$300 (-$480 to -$160) | $57,000 ($40,000 to $90,000) |
| Statin (10-14% risk) | 25,000 (14,000 to 37,000) | $2,200 ($1,500 to $2,900) | -$360 (-$590 to -$190) | $73,000 ($52,000 to $110,000) |
| Statin (5-9% risk) | 46,000 (27,000 to 70,000) | $5,500 ($3,800 to $7,400) | -$730 (-$1,200 to -$380) | $100,000 ($73,000 to $160,000) |
| Dietary advice (≥15% risk) | 79 (43 to 130) | $200 ($110 to $450) | -$0.9 (-$1.6 to -$0.5) | $2,400,000 ($1,300,000 to $6,200,000) |
| Dietary advice (10-14% risk) | 79 (44 to 130) | $260 ($140 to $580) | -$1.1 (-$1.9 to -$0.5) | $2,900,000 ($1,600,000 to $7,800,000) |
| Dietary advice (5-9% risk) | 150 (80 to 240) | $630 ($350 to $1,400) | -$2.2 (-$3.7 to -$1.1) | $3,900,000 ($2,200,000 to $10,000,000) |
| Phytosterol (≥15% risk) | 75 (36 to 120) | $570 ($350 to $860) | -$0.9 (-$1.7 to -$0.4) | $7,700,000 ($4,400,000 to $15,000,000) |
| Phytosterol (10-14% risk) | 78 (37 to 130) | $730 ($450 to $1,100) | -$1.1 (-$2.1 to -$0.5) | $9,400,000 ($5,400,000 to $18,000,000) |
| Phytosterol (5-9% risk) | 150 (69 to 250) | $1,800 ($1,100 to $2,700) | -$2.3 (-$4.2 to -$1.0) | $12,000,000 ($7,000,000 to $25,000,000) |
| NB. Values are mean and 95% uncertainty interval for health gain and costs, and median and 95% uncertainty interval for cost-effectiveness ratios. All figures are rounded to two significant figures.  ***** Where the ratio is *Dominant*, the intervention is cost-saving.  ****** Indicates interventions that are included in the optimal package when the cost-effectiveness threshold is $50,000/DALY. | | | | |
